# Behavior of potentially toxic elements from stoker-boiler fly ash in Interior Alaska: paired batch leaching and solid-phase characterization

**DOI:** 10.1007/s11356-021-15583-x

**Published:** 2021-10-23

**Authors:** Kyle P. Milke, Kiana L. Mitchell, Sarah M. Hayes, Carlin J. Green, Jennifer J. Guerard

**Affiliations:** 1 Department of Chemistry & Biochemistry, University of Alaska Fairbanks, Fairbanks, AK 99775 USA; 2 Geology, Energy & Minerals Science Center, U. S. Geological Survey, 12201 Sunrise Valley Dr., MS 954, Room 4C200, Reston, VA 20192 USA; 3 Chemistry Department, United States Naval Academy, Annapolis, MD 21402 USA

**Keywords:** Fly ash, Coal combustion products, Dissolved organic matter, Trace elements, Ettringite, Batch leaching, Physiologically based extraction test

## Abstract

Despite significant investigation of fly ash spills and mineralogical controls on the release of potentially toxic elements (PTEs) from fly ash, interactions with the surficial environment remain relatively poorly understood. We conducted 90-day batch leaching studies with paired analysis of supernatant and solid-phase mineralogy to assess the elemental release and transformation of fly ash upon reaction with aquatic media (18 MΩ cm^−1^ water and simulated rainwater). The fly ash in this study, collected from the University of Alaska Fairbanks stoker-boiler power plant, is high in unburned carbon (~20% LOI) and highly enriched in several PTEs relative to the upper continental crust. Supernatant concentrations of oxyanion-forming elements (e.g., As, Se, Mo, Sb) remained relatively low and constant, suggesting equilibrium with the solid phase, possibly ettringite [Ca_6_Al_2_(SO_4_)_3_(OH)_12_•26H_2_O], which is known to incorporate and sorb oxyanion-forming PTEs and was identified by X-ray diffraction. Synthetic precipitation leaching procedure (SPLP) results failed to capture important temporal trends. Lead and Ba supernatant concentrations consistently exceeded drinking water standards, as well as others upon exposure to simulated physiological solutions. Seven-day experiments with dissolved organic matter-isolate solutions indicated that for certain elements, liberation was influenced by carbon concentration and/or the identity of the isolate. Overall, this paired approach can serve as a model for future studies, bridging existing gaps between batch leaching and single-element mineralogical, sorption, or speciation studies.

**Supplementary Information:**

The online version contains supplementary material available at 10.1007/s11356-021-15583-x.

## Introduction

Over 70 million metric tons of coal combustion products (CCPs) were generated in the USA in 2019, constituting a major source of industrial waste (ACAA 2020; EPA 2020b). Fly ash (FA) comprises 37% by mass of CCPs generated in the USA and is often enriched in potentially toxic elements (PTEs), especially Hg, As, Cr, and Ni (ACAA 2020; EPA 2017; EPA 2019). About half of currently produced FA is reused (60% in the USA in 2019; ACAA 2020) in beneficial applications such as cement and structural fill (cf. Bartoňová 2015; Skousen et al. 2012). In interior Alaska, where coal currently generates 39% of electricity and coal reliance will continue for decades to come (McDowell Group 2018), FA is widely used as structural fill, and the remainder is disposed (ACAA 2020; EPA 2015; Lu and Rao 1971; Rettig 2011). FA can be a plausible source of PTEs to the surficial environment, especially from historically stored FA, depending on storage methods, and potentially from some reuse applications. The mineralogy, composition, and distribution of trace elements in FA are determined by the geochemical characteristics of the feed coal, combustion process, and waste handling (Hower et al. 2017). In the surficial environment, these characteristics influence the biogeochemical weathering trajectory and have important implications for the sequestration and release of PTEs. Further, aquatic systems have variable pH, Eh, and organic content and composition, all of which influence mineral weathering. Thus, assessing the fate of releasing CCPs to the surficial environment and ultimately the associated environmental and human health risks depend on the physical and chemical characteristics of both the CCPs and the aquatic systems with which they may come into contact.

Trace element volatility, condensation, incorporation into, and sorption onto particles are fundamentally controlled by the composition of source coal, combustion conditions, and the mineral or organic phases with which they are associated (Hower 2016; Xu 2004). Gaseous emissions of several trace PTEs (Sb, As, Be, Cd, Cr, Co, Pb, Mn, Hg, Ni, and Se) are regulated by the US Environmental Protection Agency (EPA) mercury and air toxics standards (MATS; EPA 2019) when emitted by power plants capable of generating > 25 MW. During combustion, elements are variably volatilized: Hg, Se, Cr, As, S, Cd, Cu, Mo, Sb, V, and Zn, in order of decreasing volatility (Ratafia-Brown 1994). Volatile elements preferentially accumulate in the FA relative to other CCPs. Lower flue gas temperatures and higher FA particle surface area tend to increase sorption and condensation of trace elements (Hower et al. 1999; Meij 1994; Vejahati et al. 2010). Further, volatile elements may accumulate on FA particle surfaces, rather than being evenly distributed within FA particles (Kukier et al. 2003). Thus, PTEs that vaporize and condense during the combustion process are often highly enriched on particle surfaces and in the smallest particles (Meij 1994; Ratafia-Brown 1994), which has a profound impact on their retention in FA and their environmental fate and transport (Iyer 2002).

Fly ash introduced into the environment, whether through reuse, large accidental release, or smaller-scale aeolian or surface water erosion from stored waste, will undergo weathering reactions that may liberate PTEs. Several studies examining large-scale accidental releases from stored CCPs have demonstrated deleterious effects on surface waters, with persistently elevated levels of PTEs in sediment pore waters and surface waters with restricted water exchange, over at least 18 months (Ruhl et al. 2010; Ruhl et al. 2009). Groundwater enriched in PTEs has been identified near CCP processing facilities in Interior Alaska (Ellis 2018). Further, significant amounts of PTE-containing particles have been documented in channels downstream of CCP releases, which may be a continued source of PTEs to the environment for decades (cf. Rivera et al. 2015; Ruhl et al. 2010; Ruhl et al. 2009).

Laboratory-based leaching studies have often been used to examine the leaching behavior of PTEs in FA. Standard leaching procedures, such as the toxicity characteristic leaching procedure (TCLP; EPA Method 1311) simulating landfill leachate, and synthetic precipitation leaching procedure (SPLP; EPA Method 1312) simulating rainwater are used to normalize the assessment of potential contaminant mobility (EPA 1992; EPA 1994). However, their short duration (18 h) raises concerns regarding their suitability for predicting long-term behavior in surficial environments, as leaching experiments of at least 90-day duration are necessary to adequately characterize elemental mobility in highly alkaline FA (Hassett et al. 2005). While a comprehensive review is beyond the scope of the current work, lab-based leaching methods have been the subject of several recent reviews (cf. Izquierdo and Querol 2012; Vejahati et al. 2010), which include studies detailing the leachability of PTEs from alkaline CCPs derived from (sub-)bituminous coals (cf. Koukouzas et al. 2011; Neupane and Donahoe 2013; Querol et al. 2001). Indeed, PTE release from FA is mediated by a variety of potential processes that are difficult to untangle, even at benchtop scale, including: dissolution, precipitation, and sorption reactions involving organic or mineral phases, which are controlled by experimental conditions, such as the solid to solution ratio, gas exchange, pH, and others. Trends in PTE releases during leaching experiments are also often difficult to interpret. For example, a recent study reported batch leaching experiments (120-h and 75-week) and 35-day column studies on the same alkaline FA derived from sub-bituminous Powder River Basin coal. The 75-week study revealed relatively consistent concentrations of Se, Mo, and Cr with time in the leachate, while Sb concentrations decreased in time in the 120-h experiment (Neupane and Donahoe 2013). In contrast, the 35-day column studies demonstrated an initial ~5 d release of Cr, Mo, and Se that accounted for nearly all cumulative release. As and Sb release kinetics were more complicated, with a slower but consistent release of As and quick release of ~25% of total Sb liberated until day 22 followed by rapid release (Neupane and Donahoe 2013). These studies illustrate the complexities of elemental leaching kinetics, which are governed by myriad competing chemical and physical processes.

Nearly all coal combusted in Alaska, including that combusted at the power plant sampled in this study, is sourced from the Usibelli Coal Mine, which annually produces 1–2 million metric tons of sub-bituminous C coal from the Suntrana Formation (seams 3, 4, 6) in the Nenana Coal Province (Schweinfurth and Finkelman 2003; UCM 2015). Prior work on Usibelli coal has determined the mineralogy, chemistry, and petrology of the feed coal, which, after low-temperature ashing, identified major kaolinite (Al_2_Si_2_O_5_(OH)_4_), minor quartz (SiO_2_), trace illite/muscovite (KAl_2_(Al,Si_3_)O_10_(OH,F)_2_), montmorillonite [(Na,Ca)_0.33_(Al,Mg)_2_Si_4_O_10_(OH)_2_·*n*H_2_O], rutile (TiO_2_), and, in some samples, plagioclase (NaAlSi_3_O_8_-CaAl_2_Si_2_O_8_), in 12 coal samples (Affolter et al. 2011). Proximate analysis for typical Usibelli coal is 29% moisture, 7% ash, 36% volatile matter, 26.5% fixed carbon, 0.2% S, and 7560 Btu lb^−1^; ultimate analysis is 70% C, 24% O, 0.3% S, 0.8% N, and 5% H, on a mass basis (UCM 2015). To our knowledge, only one study has examined Alaskan FA leaching, using samples generated by the Healy Unit 1 Power Plant (Healy, AK), a commercial coal-fired power plant burning coal from the Usibelli Coal Mine (Church et al. 1995). TCLP did not identify any associated hazards, as dissolved metal(loid)s fell below 10% of maximum contaminant levels (MCLs). However, the same study reported significant Ba release in excess of TCLP during column studies at room temperature and under freeze-thaw cycling (simulating (sub-)Arctic field conditions), highlighting the complexity of leaching processes and limitations of short-term standard leaching procedures (Church et al. 1995).

The interior Alaskan (sub-)Arctic environment is characterized by discontinuous permafrost, and typical surface water chemistry can have high dissolved organic carbon (DOC; > 10 mg L^−1^) (Ma et al. 2019; Manasypov et al. 2014; Stolpmann et al. 2021). High DOC in Alaskan surface waters is especially intriguing when considering the fate of PTEs. The composition and reactivity of dissolved organic matter (DOM), including controls on metal(loid) complexation (cf. Baken et al. 2011; Chen et al. 2018), is dependent upon its source inputs (cf. Aiken et al. 2011; Guerard et al. 2009). Interactions between DOM and certain PTEs have been explored extensively in the literature. Some metal-DOM complexes are dependent upon the extent of DOM aromaticity, while others depend on presence of negatively charged groups such as carboxylates (Aiken et al. 2011; Baken et al. 2011; Chen et al. 2018; Fujii et al. 2014). DOM may also facilitate dissolution and liberation of PTEs that would otherwise remain in their original phase or may interact with Fe (oxy)hydroxides to mobilize certain metal(loid)s (cf. Liu et al. 2011). One or more of these processes occurring simultaneously presents a complex situation for understanding the fate and mobility of CCP metal(loid)s in contact with aqueous environmental media.

Thus, we conducted 90-day batch leaching experiments, with paired supernatant analysis and solid-phase characterization to investigate elemental release and mineralogical transformation of FA upon reaction with aquatic media. The mineralogy of the source coal, unreacted FA, and leaching solid-phase leaching experiment residuals were examined using X-ray diffraction (XRD) and scanning electron microscopy (SEM). For comparison, SPLP was performed and physiologically based extraction tests (PBETs) were used to assess bioaccessibility under simulated physiological conditions, representing exposure by inhalation and oral ingestion. Further, 7-day experiments with DOM isolate solutions were performed to assess influence of elemental release by carbon concentration and/or DOM identity. Overall, this paired approach can serve as a model for future studies, bridging existing gaps between leaching studies and single-element mineralogical, sorption, or speciation studies.

## Materials and methods

### Site description

Samples examined in this study were sourced from the Atkinson Heat and Power Plant, a stoker-boiler system with a traveling grate at the University of Alaska Fairbanks (UAF). Stoker-boilers combust at a lower temperature than commercial coal-fired plants and are typically characterized by higher residual carbon (Tomei 2005). This facility has been operating since January 1964, although a new plant was completed in 2018, after the collection of samples examined in this work (UAF 2017). At the time of collection, the plant generated 3700-kW h^−1^ electricity and 100,000-lbs steam for building heat and hot water (Fig. S1). During combustion, coal pieces (passing a 5-cm grate) were fed from a storage hopper and distributed evenly across a traveling grate using a feed mechanism. Combustion occurs at temperatures in excess of 850 °C, with fine particles combusting while suspended in air and coarser particles being supported on the grate during combustion, as they travel toward a hopper where bottom ash is collected (Tomei 2005). Flue gases produced during combustion travel into the cinder reinjection hopper, where heavier particles are reinjected into the furnace. Flue gases are further treated to remove particulate matter using cyclone separators and a bag house prior to releasing gases back to the environment.

### Materials and samples

All reagents used were American Chemical Society (ACS) reagent grade or better, and all labware was acid-washed in 10% nitric acid prior to use, unless otherwise noted. The coal sample was collected at the mine prior to shipment and bag house FA was provided as collected from the Atkinson Heat and Power Plant in February 2017. The FA was dried at room temperature and homogenized prior to storage at room temperature.

### Physicochemical characterization

For detailed methodology, see Supplementary Information (SI). Elemental analysis of leachates was performed at the UAF Advanced Instrumentation Laboratory (AIL) on an Agilent 7500ce inductively coupled plasma-mass spectrometer (ICP-MS; Santa Clara, CA). Solid-phase elemental analysis of FA was primarily performed by AGAT Laboratories (Mississauga, Canada). Briefly, major elemental composition was analyzed by wavelength-dispersive X-ray fluorescence and total S and C by combustion using a LECO CHNS analyzer. Trace elemental analysis was performed using inductively coupled-optical emission spectrometry (ICP-OES) and ICP-MS following sample digestion in hot HCl, HNO_3_, HF, and HClO_4_ in Teflon vessels and sintering of any residual solids with Na_2_O_2_ and NaOH (see Table S1 for complete chemical data). Low-temperature ashing was performed to isolate the mineral fraction, followed by sequential heating to 250 °C, 400 °C, 550 °C, and 750 °C for at least 2 h in order to examine changes in mineralogy upon heating (e.g., the removal of structural waters and decomposition of carbonate minerals; see SI for details). Color was determined on dry samples using a Munsell soil color chart (Fig. S2 and Table S2; Munsell Color; Grand Rapids, MI).

XRD analysis was performed on (1) each unreacted coal and FA, (2) each unreacted coal and FA subjected to low- and high-temperature ashing (Figs. S3-S4 and Tables S3-S4), (3) solid-phase residuals from leaching experiments at selected time points (1 h, 2 days, 7 days, 14 days, 28 days, 90 days; Fig. S5, Table S5), and (4) experimental replicates of 14-day 18 MΩ experiment samples as well as a second analysis of one of the replicates (Fig. S6, Table S6; details in SI). All FA samples were spiked with a corundum (Al_2_O_3_) internal standard at 15% wt/wt to quantify amorphous material (Brindley and Brown 1980), micronized, sieved, and analyzed using a PANalytical X’Pert PRO (Almelo, the Netherlands) automated powder diffractometer. Quantitative mineral-phase identification was performed using the Rietveld module of PANalytical X’Pert HighScore Plus software (Almelo, the Netherlands; version 4.8.0.25518) and standard reference patterns from the Inorganic Crystal Structure Database (ICSD; ICSD 2013). Depending on the peak position and peak overlaps, analysis of XRD patterns with Rietveld refinements typically has detection limits of 1% (Smith et al. 2013), but some mineral phases are reported at values near and below this detection limit based on diagnostic peak positions and/or phase confirmation using SEM. SEM was used to examine particle morphology and confirm the presence of minerals identified by XRD. SEM was performed on unreacted FA prepared as a polished puck and as a powder dispersed on tape (details in SI; Figs. S7 and S8).

Multi cross-polarization magic angle spinning (multiCP-MAS) ^13^C nuclear magnetic resonance (NMR) (Johnson and Schmidt-Rohr 2014) was used to characterize the organic components of the CCPs after a series of hydrofluoric acid washes to remove paramagnetics (Ehlers et al. 2010). The analysis was performed using a Bruker Avance III 600 MHz spectrometer (Billerica, MA) at 150 MHz ^13^C frequency, using a Bruker 4 mm MAS probe to analyze the samples, which were diluted with KBr to prevent arcing. A total of 40,000 scans were collected using a spin rate of 15 kHz. Spectra were processed using Topspin 3.5.7. To determine the semiquantitative distributions of various functional groups, spectra were integrated between corresponding chemical shift regions (Cawley et al. 2013).

### Standard leaching experiments

SPLP was performed according to EPA method 1312 (EPA 1994). Supernatant alkalinity, pH, electrical conductivity, and anions were measured using standard methods as described in the SI. Physiologically based extraction tests (PBETs) mimicking the fasting stomach and alveolar conditions were performed using established protocols (see SI for details; Table S7) (EPA 2012; Stefaniak et al. 2006). Elemental analyses for the SPLP and PBETs were performed using ICP-MS.

### Leaching experiments

Long-term batch leaching experiments were performed with 2 g of FA reacted with 40 mL of solution per polypropylene centrifuge tube on an end-over-end rotator, operating at 8 rpm. Centrifuge tubes were not capped and instead covered with a layer of Parafilm held in place by a rubber band to enable gas exchange. Leaching media included a >18 MΩ cm H_2_O control (18 MΩ) and simulated rain water (RW) (Koch et al. 1986). Triplicate samples were sacrificed at 13 time points: 0 h (method blank), 1 h, 12 h, 1 day, 2 days, 3 days, 5 days, 7 days, 10 days, 14 days, 21 days, 28 days, and 90 days. To terminate the experiment, samples were centrifuged prior to filtering to 0.45 μm, pH measurement, and acidifying the supernatant for ICP-MS analysis. The solid residue was rinsed with >18 MΩ cm H_2_O prior to freeze-drying (FreeZone Plus 12 Liter Cascade, Labconco; Kansas City, MO). The percent released was calculated by dividing the mass of each element in the 40-mL supernatant solution by the total mass of that element in the 2-g solid in the tube and multiplying by 100% (Table S8).

Seven-day batch experiments were performed with FA and reconstituted DOM isolate solutions to simulate interactions with aquatic organic media. Suwannee River fulvic acid (SRFA) and Pony Lake fulvic acid (PLFA) were obtained from the International Humic Substances Society (St. Paul, MN) (Averett et al. 1994; Cawley et al. 2013). An AK organic matter solid-phase extract (AKSE) was isolated according to Dittmar et al. (2008) from a thermokarst lake (55 mg C L^−1^) in Fairbanks, AK, in June 2017 and has been previously characterized (Gagne et al. 2020). DOM solutions were prepared at 0, 5, 10, 20, 50, and 100 mg C L^−1^. Duplicate samples of 2 g of FA were reacted with 40 mL of each DOM solution for 7 days in the dark at room temperature, without redox control in an end-over-end rotator (8 rpm). Reactions were terminated by centrifugation, filtration, and acidification prior to dilution and analysis by ICP-MS. Only major or toxic elements (Ca, Fe, Pb, Mo, Cr, Se, Sb, As, Cd) were targeted for analysis due to cost and capacity limitations.

### Geochemical modeling

Mineral dissolution reactions were used to model the saturation of barite, gypsum, and ettringite. As sulfate concentrations by ion chromatography were unavailable, this was done with the assumption that the sulfate ion concentrations would be equivalent at equilibrium and using standard solubility products (Skoog et al. 1996). Ettringite has a variety of published solubility products (Hampsoim and Bailey 1982; Jones 1944; Myneni et al. 1998) that can be affected by oxyanion substitution (Baur and Johnson 2003; Chrysochoou and Dermatas 2006; Perkins 2000). Thus, the ettringite stability zone was calculated using the highest and lowest pH and concentration of Al measured in sample solutions with the highest and lowest reported solubility products, creating the widest possible stability zone (additional details in SI).

## Results

### Physical characterization

**Fig. 1:**
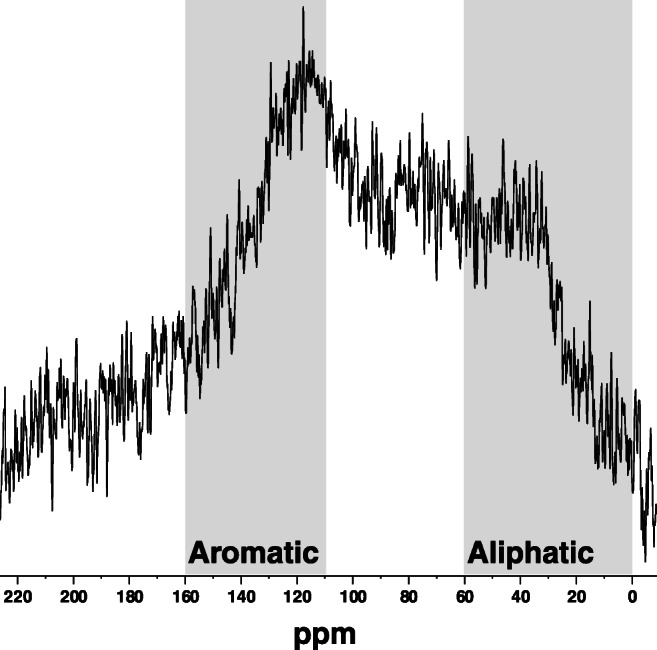
Solid-state multiCP-MAS ^13^C NMR of FA. Gray regions (0–60 ppm, 90–160 ppm) broadly represent denotation of aliphatic and aromatic functional groups, respectively

As expected, the stoker-boiler FA contained high unburned carbon content, as indicated by a dark gray-black color, 22% loss on ignition (LOI), and 19% total carbon (Table 1). This value is similar to the values reported previously for stoker-boiler systems (Bartoňová 2015; Mardon et al. 2008) but much higher than the average 3% unburned carbon for all coal ashes (Bartoňová et al. 2012). While the high LOI renders this FA unsuitable for reuse in cement (LOI must be < 6%; ASTM 2019), the high carbon content enabled nearly quantitative solid-state multiCP-MAS ^13^C NMR. Integration of chemical shift regions identified a heterogeneous organic composition composed of a large percentage of olefinic/aromatic groups (42%) but also aliphatics, substituted aliphatics, and carbonyls (Fig. 1 and Table 1). The FA was also enriched in several PTEs relative to average crustal abundance (Rudnick and Gao 2006). Depletion or enrichment factors for Cr, As, Se, Mo, Sb, and Pb were 0.9, 26, 240, 10, 67, and 8.4, respectively (Table 1). This is consistent with prior studies that identified PTE enrichment in FAs (Meij 1994).

**Table 1:** Composition of FA used in this study

Major elements			Trace elements			Carbon composition	
Elements	wt.% ^*a*^	Enrichment rel. to UCC^*c*^	Elements	mg kg^−1^	Enrichment rel. to UCC^*c*^	Moieties and chemical shift (ppm)	Rel. integration (%) ^*e*^
C^*b*^	18.6 ± 0.1	---	As	124.5 ± 0.7	26	Aliphatic I (0–45)	19
LOI	21.7 ± 0.1	---	Ba	4320 ± 20	7	Aliphatic II (45–60)	8
Al_2_O_3_	11.4 ± 0.2	0.7	Co	27.4 ± 0.6	1.6	Sub. aliphatic (60–90)	15
CaO	26.9 ± 0	7.5	Cr	85	1	Acetal-aromatic (90–160)	42
Fe_2_O_3_	9.78 ± 0.01	1.7	Cu	840 ± 20	30	Carboxyl (160–190)	9
MgO	5.25 ± 0.01	2.1	Hg	3.6 ± 0.1	72	Carbonyl (190–220)	6.5
S^*b*^	2.12 ± 0.08	34	Mn	2670 ± 10	3		
SiO_2_	18 ± 1	0.3	Mo	11.4 ± 0.3	10		
			Pb	140 ± 10	8		
			Sb	27 ± 2	67		
			Se	22	244		
			V	160 ± 3	2		
			Zn	237 ± 8	4		
			Σ REEs^*d*^	217 ± 1	1		

^a^Total elemental analysis performed by wavelength-dispersive X-ray fluorescence for major elements (expressed as oxides) and by a 4-acid digest followed by peroxide sinter of residue for trace elements (expressed in mg kg^−1^), except where noted. Error is reported as the standard deviation of duplicate measurements. Complete elemental analysis and quality assurance information are available in Table S1

^b^Total sulfur and carbon measured by combustion

^c^Upper continental crust (UCC) (Rudnick and Gao 2006)

^d^Sum of yttrium and rare earth elements (atomic numbers 39, 57-60, 62-71)

^e^Relative integrations of multiCP-MAS 13C NMR spectra

### Mineralogy of coal and unreacted fly ash

Healy coal was composed of 80+% organic material based on LOI and mass loss upon low-temperature ashing (LTA; Fig. S2 and Table S2). The XRD-detectable minerals in the coal were major quartz and kaolinite with minor illite (Fig. S3, Table S3), with major bassanite after LTA attributed to the oxidation of organic matter (Allen et al. 1986). This is consistent with a prior study of 12 Usibelli coal samples, which did not detect bassanite (CaSO_4_·1/2H_2_O) upon ashing, but identified major kaolinite, minor quartz, and trace or sporadic plagioclase, illite/muscovite, montmorillonite, and rutile (Affolter et al. 2011). The same study also examined coal from other major US coal basins and identified similar mineralogy with quartz, a variety of clay minerals, sulfides, carbonates, oxides, and sulfates in the coals of various rank from the Illinois, San Juan, Appalachian, and Powder River coal basins (Affolter et al. 2011). Thus, Alaskan coal has similarities to other coals, with the main exception that sulfides and sulfates were not detected. At combustion temperatures, some minerals melt (e.g., kaolinite) and, depending on conditions, can form amorphous glassy material or other neoform silicate minerals, making the FA mineral assemblage distinct from, but related to, that of the original coal.

**Fig. 2:**
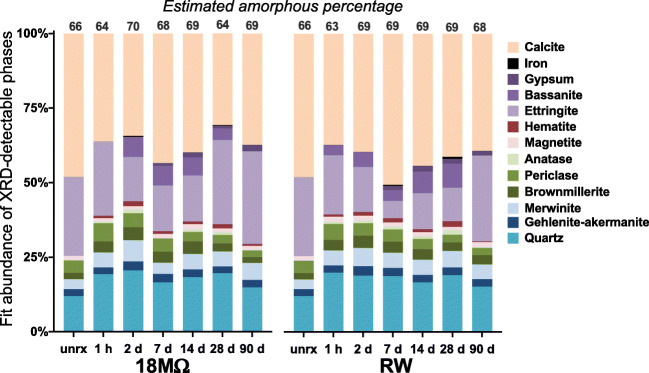
Mineralogy of unreacted FA and solid-phase leaching residuals as determined by Rietveld refinements. Estimated amorphous percentage was determined using corundum as an internal standard. Plotted mineral abundance is normalized to 100% for XRD-detectable phases

XRD and SEM examination of unreacted FA yielded consistent results; the FA was found to contain both quartz and several minerals that form under Si-poor conditions: gehlenite-akermanite (Ca_2_Al(Si,Al)_2_O_7_-Ca_2_MgSi_2_O_7_), merwinite (Ca_3_Mg(SiO_4_)_2_), and brownmillerite (Ca_2_(Fe,Al)_2_O_5_; Fig. 2; Deer et al. 1992). The co-occurrence of these minerals indicates that the assemblage was not at equilibrium, likely explained by the precipitation of neoformed minerals from Si-poor melt produced during combustion and entrained quartz residual from the source coal. Significant amounts of calcite (CaCO_3_) and ettringite (Ca_6_Al_2_(SO_4_)_3_(OH)_12_·26H_2_O) and trace amounts of periclase (MgO) and magnetite (Fe_3_O_4_) were also identified. The presence of ettringite is especially important, since ettringite can influence the release of some oxyanions in CCPs under high pH conditions (Hassett et al. 2005; Myneni et al. 1998).

This mineral assemblage is largely consistent with a prior study, which examined Usibelli coal–derived CCPs from a commercial coal-fired power plant in Healy, AK (Affolter et al. 2011). Notable exceptions include the much higher fraction of calcite and ettringite in the UAF FA, and several trace minerals identified in the Healy FA (plagioclase, pyroxenes, mullite, lime, anhydrite) were not detected in the UAF FA. As the raw coal is from the same source, these differences are attributed to differing combustion processes, firing temperatures, and, possibly, pollution controls at the two facilities. Alaskan FA and FA generated from sub-bituminous coal from the Powder River Basin have similar mineralogy, all containing merwinite, gehlenite-akermanite, and only trace mullite, when compared with FA generated in commercial coal-fired power plants from other parts of the USA burning other rank coals (Affolter et al. 2011). Prior work on an unspecified stoker-boiler FA indicates primarily mullite, quartz, and hematite but highlights the high fraction of amorphous material associated with the stoker-boiler FA relative to the National Institute of Standards and Technology (NIST) 1633a, which was generated from PA and WV coals (NIST 1985). Undoubtedly, the differences between Alaskan FA and FA generated elsewhere are due to the coal characteristics and combustion process and serve to highlight the unique mineralogy of the FA examined in this study.

### Mineralogical changes during leaching

Fly ash mineralogy did not change significantly upon leaching and contained quartz, a suite of minerals indicative of a Si-poor formation environment (gehlenite-akermanite, merwinite, brownmillerite), oxides (periclase, magnetite, hematite (Fe_2_O_3_), anatase (TiO_2_)), elemental iron, calcite, and sulfates (ettringite, gypsum, bassanite; Fig. 2). Corundum was added as an internal standard, and the results indicate that all samples contain significant amorphous material (63–70%), likely composed of residual organics and amorphous glassy material formed during combustion. Semiquantitative SEM energy-dispersive spectroscopy (EDS) analysis of homogeneous, presumably amorphous, spheres indicated they contain significant amounts of O-Ca-Si-Al-Fe-Mg-Ti and intermittent, trace amounts of Na, K, and Mn.

The replicate analyses (Table S6) estimated a range of 68–70% amorphous material, suggesting the fits are relatively precise. The unreacted FA fit contained 66% amorphous material, which decreased to 63–64% after 1 h of leaching, potentially indicating the quick dissolution of some soluble amorphous material (Fig. 2). However, the fraction of amorphous content increases by the 2-day time point to a value higher than the unreacted FA (68–70% amorphous content in fit), which is relatively consistent for the remainder of the experiment. The exception to this is the 28-day 18MΩ residual fit, which has a value similar to the 1-h residual (64% amorphous fit). Together, these results seem to indicate the initial dissolution of soluble amorphous material followed by the reprecipitation of amorphous material to an extent slightly greater than present in the unreacted FA.

However, small mineralogical changes are evident after 1 h of leaching. Most notably, the significant dissolution of calcite within 1 h, which is consistent with the high concentrations of Ca in solution. Calcite abundance fluctuates, reaching minimum abundances at 2 and 28 days in both the 18 MΩ and RW experiments. Ettringite abundance also decreases, although more slowly, with minimum abundance between 2 and 28 days for both experiments and increasing by 90 days to final abundances slightly higher than in the unreacted FA. Also, minor decreases in a few silicate and oxide minerals indicate the slow dissolution of quartz, brownmillerite, and periclase throughout the experiment. These dissolution reactions produce hydroxide ions and may help to maintain the high pH of the system over long timescales.

New minerals also precipitate during leaching, as revealed by XRD (Fig. 2). Hematite is evident after 1 h and remains as a consistent trace constituent for the rest of the experiment. Bassanite, a metastable calcium sulfate, appears quickly (within 2 days and 1 h in the 18 MΩ and RW experiments, respectively) and is consistently present except in the 90-day solid-phase residuals. Gypsum, a likely transformation product of bassanite, first appears visually at 2 days as a broad hump at 10.5 °2θ and begins to be included in fits at 7 days. This gypsum peak progressively sharpens with increasing leaching time, indicating an increase in crystallinity over time. As has been reported in similar systems, thermodynamic modeling indicates that gypsum and by extension bassanite are undersaturated but are clearly present (Myneni et al. 1998).

### Standard leaching experiments

**Fig. 3:**
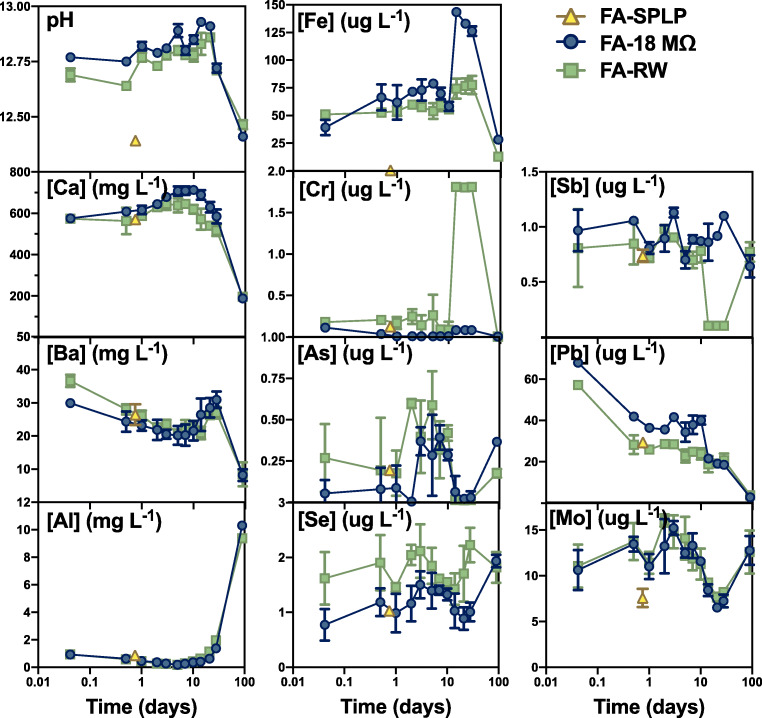
Supernatant concentrations of selected elements as a function of time and SPLP results

SPLP results indicate UAF FA is generally not hazardous, consistent with previous TCLP results (Church et al. 1995). The only exceptions are Ba, Pb, and fluoride in the leachate, which exceed US EPA drinking water standards (DWS) tabulated in Table S7 (EPA 2018). Concentrations measured in SPLP leachates are generally in good agreement with the long-term leaching results at similar reaction times (Fig. 3).

Physiologically based extraction tests indicate variable liberation of PTEs (up to 37%) under simulated gastric conditions (Table 2). Gastric PBET supernatant concentrations of Pb, As, and Sb exceed the EPA DWS by 34×, 14×, and 10×, respectively (EPA 2018). Simulated lung fluid solubilized a smaller fraction of PTEs relative to gastric extractions (except for Se, with 28% liberation), consistent with previous studies (Knight et al. 2017; Schaider et al. 2007), but still exceeded EPA standards for As and Se by 1.8× and 1.2×, respectively. Taken together, the SPLP and PBET results indicate that additional examination of this FA is warranted because of regulatory exceedances and significant bioavailability of PTEs in this FA.

**Table 2:** Gastric and lung physiologically based extraction tests for FA expressed as final supernatant concentrations and as percentage of total released

	Gastric		Lung	
Element	μg L^−1^	%	μg L^−1^	%
Cr	50 + 2	5.8 + 2	11 + 1	1.3 + 1
As	140 + 10*	11.0 + 3	18 + 4*	1.5 + 3
Se	39 + 1	17.8 + 7	62 + 4*	28 + 2
Sb	61.5 + 7*	23 + 2	14 + 1*	5.3 + 5
Pb	510 + 40*	37 + 4	BDL	

Values in parenthesis represent the standard deviation of triplicate measurements. Starred values represent values above EPA DWS standards (EPA 2018)

### Long-term batch experiments

The pH increased to ~12.7 within the first hour and then was relatively consistent, with a slight decrease at 90 days (pH is 12.4 and 12.5 for 18MΩ and RW leachates, respectively; Fig. 3). Overall, only a small fraction of the elements in the FA were solubilized in the 90-days leaching experiment (< 0.1% for most elements; Table 3), consistent with the small mineralogical changes observed, but lower than typically reported (Izquierdo and Querol 2012). This suggests either slow dissolution kinetics, precipitation of secondary phases, and/or sorption/sequestration of ions released.

**Table 3:** Percent leached from solid phase

	18 MΩ			RW		
Element	1 h	7 d	90 d	1 h	7 d	90 d
Ca	6	7	2	6	7	2
Ba	14	9	4	17	10	4
Fe	0	0	0	0	0	0
Al	0.03	0.01	0.3	0.03	0.01	0.3
V	0	0.01	0.02	0	0.01	0.02
Cr	0	0	0	0	0	0
Co	0.09	0.1	0.04	0.08	0.09	0.05
Cu	0.03	0.04	0	0.02	0.01	0
As	0	0.01	0.01	0	0.01	0
Se	0.07	0.1	0.2	0.1	0.1	0.2
Mo	2	2	2	2	2	2
Sb	0.07	0.07	0.05	0.06	0.05	0.06
Pb	1.0	0.5	0.04	0.8	0.4	0.06

More than 1% of total Ca and Ba were released throughout the 90-day experiments (Table 3). Supernatant Ca concentrations were about 600 mg L^−1^ after only 1 h, indicating that about 6% of the total calcium was highly labile. Aqueous Ca gradually increased to day 7 or 14 to a maximum of about 700 mg L^−1^ and then decreased to about 200 mg L^−1^ at 90 days (Fig. 3). Barium had the largest percentage released from the solid phase relative to other elements during the leaching experiments (4–17%). Barium leachate concentrations (initially ≥ 30 mg L^−1^) decreased until 14 days before a slight increase at 21 days and decreased steeply to a final concentration of 8 mg L^−1^ (Table S7). These relatively high Ba concentrations in solution exceeded the EPA DWS MCL of 2 mg L^−1^ throughout both experiments (EPA 2018). Aluminum concentrations in solution were initially low (< 1 mg L^−1^) until 28 days but increased to up to 10 mg L^−1^ by 90 days. Iron leachate concentration was relatively consistent at roughly 50–75 μg Fe L^−1^ until day 10 for both experiments, but then Fe concentration in the 18MΩ supernatant increased up to 130 μg Fe L^−1^ at 14–28 days before decreasing below initial values by 90 days.

Among the minor and trace elements, most notable was that high concentrations of Pb were measured after just 1 h (up to 68 μg L^−1^; nearly 1% total Pb; Fig. 3 and Table 3) and steadily decreased in both experiments to under 4 μg L^−1^ by 90 days. Nearly all Pb concentrations (except 90 days) exceeded EPA DWS (EPA 2018), and the 1-h 18MΩ supernatant also exceeded the nominal EPA Freshwater Criteria Maximum Concentration (FW CMC, [Pb] < 65 μg L^−1^; EPA 2020a). Molybdenum concentrations were relatively consistent over time, ranging from 6 to 15 μg L^−1^ (representing up to 2.3% of total Mo). Chromium concentrations liberated from FA were quite low (< 0.2 μg L^−1^) for almost all experiments, but with a spike in the RW up to ~1.7 μg L^−1^ during 14–28 days. Selenium, Sb, and As leaching trends were similar in that only low, invariant concentrations of these elements were liberated (> 3 μg L^−1^).

### FA-DOM leaching experiments

**Fig. 4:**
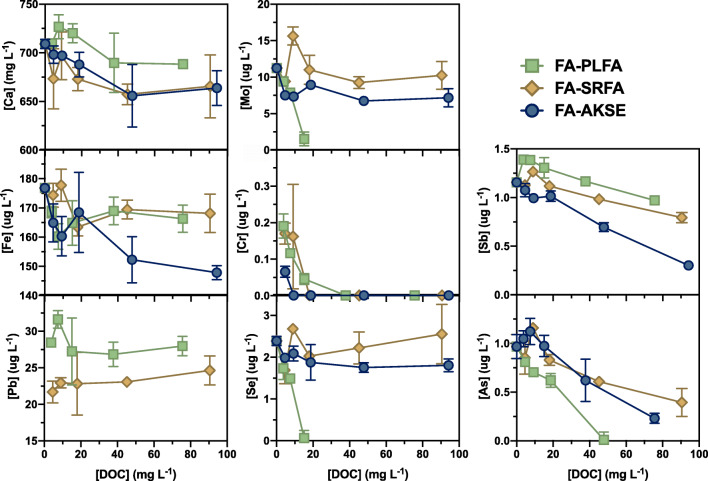
Supernatant concentrations of selected elements as a function of dissolved organic carbon concentration ([DOC]) in 7-day experiments

Closed batch leaching experiments with DOM revealed significant color loss, based on the visual observation that the initially yellow hue of the DOM isolate solutions was no longer observable after the supernatant was filtered at the end of the experiment. Similar to the long-term batch experiments, the pH of all solutions increased from acidic (pH = 3.4–4.9) to pH = 12.5–13.3 after 7 days. Control experiments (i.e., no DOM) were generally similar to 7-day metal(loid) supernatant concentrations from the long-term leaching experiments (Figs. 3 and 4), with the exception of Fe. Cadmium leachate concentrations were below detection limit (BDL) for all DOM solutions at all carbon concentrations, and several other elements (e.g., Cr, Se) also had some leachate concentrations BDL.

Several trends were observed with PTE supernatant concentrations with variable initial DOC concentration ([DOC]) and DOM isolate. Calcium and Fe concentrations were invariant for all [DOC] and DOM isolate (< 20% variation and overlapping error bars). Lead supernatant concentrations were invariant to [DOC], but higher Pb concentrations were observed in PLFA relative to SRFA. Several elements were impacted by both the [DOC] and DOM isolate, but not always in the same way. Se and Mo were largely invariant to [DOC] for the SRFA and AKSE, but their supernatant concentration decreased dramatically as [DOC] increased in the case of PLFA. In contrast, As, Sb, and, especially, Cr concentrations decreased with increasing [DOC], but the effect was more pronounced in the AKSE relative to the other two DOM isolates.

## Discussion

Mineral transformation, solution chemistry, and, by extension, PTE release are controlled by a variety of environmental conditions, including the leaching solution composition, gas exchange rates, pH, FA characteristics (surface area, particle size, organic moieties, composition), and heterogeneity of elemental distribution within FA particles. These conditions give rise to a variety of competing processes, which can occur simultaneously in aquatic systems, such as mineral dissolution or precipitation and sorption to organic or mineral phases. Thus, characterization of both leached supernatant chemistry and solid-phase transformations is essential for understanding the long-term stability of phases controlling PTE release.

### Standard procedures fail to capture PTE releases at short and long timescales

Elemental concentrations measured by the SPLP and ~18-h leaching time points were typically in good agreement. Barium and lead were the only elements to exceed the EPA DWS over the 90 days, and these also exceeded the DWS during the SPLP experiment. However, the SPLP fails to capture important temporal complexity of elemental release, which may be a critical component of risk evaluation (Fig. 3). For example, the SPLP was not adequate to predict longer-term releases since there are striking differences between the SPLP and 90-day leachate concentrations. Especially since several PTEs fail to reach equilibrium within 90 days, longer experiments would be necessary to assess release, as previously noted (Hassett et al. 2005). Another shortcoming of the SPLP revealed by this experiment was that it failed to capture immediate releases of PTEs that could result in acute toxic effects, such as Pb, which reached concentrations of up to 68 μg L^−1^, in excess of the nominal EPA FW CMC, an acute toxicity guideline, within just 1 h. In sum, our results support that standard leaching procedures may not be suitable for predicting the short- and long-term behavior of PTEs in surficial environments.

### Unreacted FA characteristics

Unreacted FA is a complex matrix composed of residual organics, amorphous glassy particles, and XRD-detectable calcite as well as a variety of silicate, oxide, and sulfate minerals (Table 1, Fig. 2). XRD reveals only minor mineralogical changes within 90 days, consistent with the relatively low rates of elemental liberation in this study (Table 3), especially relative to other similar studies (Izquierdo and Querol 2012). Indeed, the abundance of most silicate and oxide minerals in FA are invariant with time, except periclase, which decreased slightly in abundance over time. While most silicate and oxide dissolution reactions produce hydroxides, they may be kinetically limited or thermodynamically unfavorable when the solution pH is high but may become important at longer timescales. Elemental iron is intermittently identified throughout the leaching experiment and in unreacted FA by SEM as tiny inclusions, which may limit lability (Fig. S8). Hematite is also consistently evident in the leached FA at trace levels beginning at 1 h, which is consistent with relatively low Fe in solution.

Perhaps the high residual carbon is one of the most unique characteristics of the UAF FA. Our results are not inconsistent with prior studies indicating the presence of graphitic and fullerene-like carbons in FA (cf. Bartoňová 2015; Bartoňová et al. 2007; Hower et al. 2017). Indeed, the solid-state multiCP-MAS ^13^C NMR spectra of FA was only possible because of the high amount of unburned carbon. Unburned carbon has previously been identified as an important sorbent for PTEs released during leaching (Bartoňová et al. 2012) and may explain the overall low fractions of elements liberated in this study (Table 3).

### Mineralogical controls and leaching behavior

**Fig. 5:**
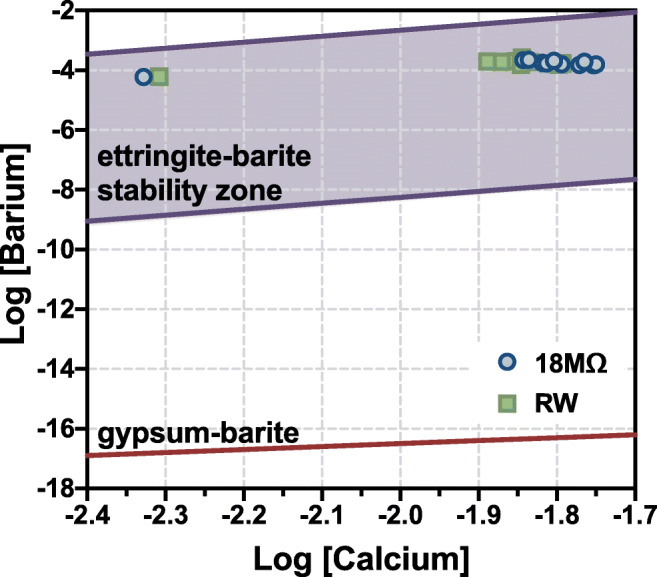
Supernatant chemistry within the context of barite, gypsum, and ettringite stability. Ettringite stability zone shown based on variations in published solubility products and experimentally measured pH and Al concentration ranges

Within 1 h of exposure to aquatic media, significant calcite dissolution was observed and may be a major contributor to increasing the system pH through bicarbonate formation, consistent with high Ca (~600 mg L^−1^) measured in solution after 1 h. Supernatant Ca steadily increases until 7 days, indicating continued dissolution Ca-containing phases. Sources of Ca to solution include calcite (which reaches a minimum abundance on day 2), ettringite (which is low between 2 days and 14 or 28 days for 18 MΩ and RW, respectively), brownmillerite after 14 days, and, potentially, Ca-containing amorphous material. Dissolved Ca decreases to ~200 mg L^−1^ at 90 days, potentially indicating both the exhaustion of readily soluble Ca sources and the (re)precipitation of Ca-containing phases: calcite, ettringite, and calcium sulfates (Catalano et al. 2012; Neupane and Donahoe 2013). Although thermodynamic modeling favors the precipitation of ettringite and barite over gypsum, both metastable bassanite and gypsum form during the leaching experiments. Figure 5 shows that experimental data lie within the barite-ettringite stability zone (the zone is defined by the maximum and minimum measured pH and [Al^3+^], see SI for details), indicating these phases are in equilibrium. Despite thermodynamic predictions, bassanite is detected by XRD as early as 1 h and disappears by 90 days consistent with the metastability of this phase, and the more stable gypsum appears after 7 days. The formation of thermodynamically unfavored phases may be due to supersaturated solutions that could form in pore spaces, in neoformed amorphous phases near weathering surfaces, or other microenvironments where reaction rates are fast relative to diffusion rates (Putnis 2009; Ruiz-Agudo et al. 2016). The higher abundance of calcium sulfate phases during the middle time points of the leaching experiments may be explained by low ettringite abundance. The decreasing dissolved Ba concentrations may indicate a labile pool of Ba that is progressively depleted during the leaching experiments by barite precipitation or incorporation of Ba ions into other phases, such as calcite. Although this is not favored based on ionic radii (Shannon 1976) based on the + 15% from Goldschmidt rules of substitution for ionic solids (Goldschmidt 1954), there is experimental evidence supporting the substitution of Ba for Ca in minerals (Ba for Ca in apatite; Lucas et al. 1990). A Ba-O-S-Al-Fe–containing particle was identified by SEM and tentatively identified as a mixed barite phase (Fig. S7d).

The apparent initial decrease in amorphous material within the first hour of reaction with aqueous media may be attributed to the dissolution of readily soluble amorphous material. However, within 2 days, the amorphous content increases to higher levels than were present in the unreacted material. This, along with the solution data, indicate that mineral transformations are occurring and neoformed minerals and amorphous material are likely forming.

Ettringite is identified in unreacted and all leached FA samples. It reaches a minimum abundance during the middle of both leaching experiments but is slightly more abundant at 90 days than in the unreacted FA (Fig. 2). Ettringite formation is favored at high pH (>10.7; Myneni et al. 1998) and is likely an important control on constituent ions (Ca^2+^, Al^3+^, SO_4_^2−^, OH^−^) and oxyanion-forming PTEs (Fig. 5). Ettringite allows extensive incorporation of potentially toxic oxyanions, which can substitute for sulfate between column structures (e.g., B, Cr, Se, As) or associate with surface sites (e.g., Sb, As; Baur and Johnson 2003; Chrysochoou and Dermatas 2006; Guo et al. 2017; Hassett et al. 2005; Leisinger et al. 2010; McCarthy et al. 1991; Myneni et al. 1997; Perkins 2000; Zhang and Reardon 2003). Similar prior work with a slightly lower pH (~11.3–10.5)10.5), below ettringite stability, reported higher PTE supernatant concentrations, which were quickly released and remained in solution for the remainder of the experiment (Neupane and Donahoe 2013). The lower PTEs in solution in the present study may be due to ettringite formation, which would not have been as favorable in the prior study.

Oxyanion-forming PTE concentrations were consistently low and constant during both leaching experiments, indicating equilibrium with the solid phase, either due to recalcitrant hosts or high affinity for minerals or organic material in the system. Ettringite may act as a sink for oxyanion-forming PTEs, especially since some of the highest supernatant concentrations of oxyanions were found when ettringite abundance is low (2–28 days; especially As and Mo). Tetrahedral oxyanions substitute for sulfate in ettringite structure, but larger As and Sb oxyanions instead sorb to the surface (Hassett et al. 2005; Myneni et al. 1997). The published ettringite oxyanion affinity series (B(OH)_4_^−^ > SeO_4_^2−^ > CrO_4_^2−^ > MoO_4_^2−^; (Zhang and Reardon 2003)) corresponds well with the average measured supernatant concentrations in this study (Fig. 3), except for Cr. For example, Mo, which is often considered a good indicator element of the environmental leaching potential of CCPs, has the highest supernatant concentration and largest percentage liberated from the solid phase despite having the lowest abundance in the unreacted FA, which is consistent with a lower rate of incorporation in ettringite due to the larger oxyanion size (Izquierdo and Querol 2012). However, causal relationships are difficult to identify since competing processes, especially sorption to organic material and incorporation into other minerals, may also influence supernatant concentrations.

Probing specific PTE-organic interactions is beyond the scope of the current study, but residual organic matter may also influence the behavior of PTEs, including oxyanion-forming PTEs. For example, increased proportions of elemental Se have been noted to increase with increasing C content, which may limit Se mobility (Luo et al. 2011). Given the low supernatant concentrations of Se observed during leaching, interaction with organics cannot be ruled out, especially since Se is volatilized during combustion, likely enriched on FA particle surfaces, and, thus, anticipated to be highly mobile.

### Interaction of DOM with CCPs

DOM-metal(loid) interactions may either facilitate or inhibit dissolution, complicating the processes described above (Aiken et al. 2011). Interactions with metal(loid)s can occur through complexation, either directly between an element and DOM as may be the case for cationic forms or via ternary complexation with elements such as Fe for oxyanions (Martin et al. 2017; Peel et al. 2017). The loss of color of the solutions after 7 days suggests a decrease in the freely dissolved DOM in the supernatant (there was not enough sample to submit for DOC measurement). This most likely indicates adsorption of the DOM onto CCP particulates, which are good sorbents for DOC (Wang et al. 2009), especially for CCPs with significant amounts of unburned carbon (Wang et al. 2005). This may most affect elements preferentially adsorbed to sites such as unburned carbon or volatile metal(loid)s associated with CCP particle surfaces and most access to surficially associated DOM. Released elements could stay complexed to the adsorbed DOM rather than exchanging with the bulk aqueous phase and may help to explain sharp decreases in supernatant concentrations for some elements (Cr, As, Sb; Fig. 4). However, a careful monitoring of DOC and metal(loid) speciation over time would be required to identify if this were actually the case. Other processes of DOM removal from solution via aggregation, precipitation, or transformation of DOM chromophores to the point of being rendered colorless also cannot be eliminated.

For a few metal(loid)s, the source of DOM isolate made a difference in its resulting supernatant concentration. This was especially observed for Se, where supernatant concentrations remained constant or increased for SRFA and AKSE, but decreased for PLFA with increasing DOC. AKSE was previously characterized by optical properties as being terrestrial in character, more similar to SRFA than PLFA (Cawley et al. 2013; Gagne et al. 2020). For some metal(loid)s, terrestrial characteristics such as aromaticity may be a contributing factor in metal(loid) complexation, such as Pb, Zn, and Cd, and, to a lesser extent, Cu (Baken et al. 2011; Chen et al. 2018). Ferric iron has also been associated with more aromatic DOM (Fujii et al. 2014) and might enhance supernatant concentrations for predicted oxyanions through ternary complexation. However, the inconsistent behavior of oxyanion-forming elements does not support that this is the primary mechanism controlling their behavior, or it could simply be that these oxyanions are not able to compete with the abundant hydroxide for complexation sites. Further study is obviously needed to resolve this issue, but this is the first study to our knowledge to explore the source-dependency of DOM mitigating metal(loid) release from CCPs.

### Lead and barium exceed EPA drinking water standards

Lead and Ba were the only elements to consistently exceed EPA drinking water standards in the supernatant during leaching of FA. Labile pools of both Pb and Ba must exist, since both elements reach their highest concentration within 1 h before generally decreasing with increased leaching time. Barium availability in natural systems is typically limited by the low solubility of barite or witherite, with the highest reported concentrations in well waters 10 ppm (ASTDR 2007), making the concentrations observed here quite remarkable. Lead leaching concentrations are consistent with a labile pool of Pb, possibly due to condensation on the surface of FA particles, as has been previously reported (Izquierdo and Querol 2012). In contrast, other studies observed very little Pb mobility (Izquierdo and Querol 2012). Decreases in Pb and Ba concentrations may be due to precipitation of insoluble minerals, such as Pb-phosphates, barite, or witherite (BaCO_3_); only a single Ba-containing particle was detected by SEM (Fig. S7d). This may not be surprising, since these elements were present in trace amounts (140 mg Pb kg^−1^ and 4320 mg Ba kg^−1^) and, if present as minerals with a high mass fraction of Pb or Ba, they would be present in very low abundance. Perhaps a more likely mechanism of sequestering these cations would be sorption to or coprecipitation with existing or neoformed minerals in FA, such as calcite, amorphous material, or iron oxides. While extensive substitution of Pb^2+^ and Ba^2+^ for 6-coordinate Ca^2+^ in calcite is not favored based on Goldschmidt’s rules for ionic solids (Goldschmidt 1954; Shannon 1976), literature does report this substitution could be an important mechanism of sequestration (Lucas et al. 1990; Andersson et al. 2014; Monasterio-Guillot et al. 2020). Further, Pb also has a high affinity for aromatic moieties (Chen et al. 2018), which were abundant based on the NMR (Fig. 1) and could partly explain both the removal of Pb from solution (Fig. 4) and that Pb supernatant concentrations are lower upon reaction with SRFA relative to PLFA, since SRFA has more aromaticity (Cawley et al. 2013).

Thus, this FA does pose potential cause for environmental and human health concerns related to Pb and Ba. If released to the surficial environment, the solid to solution ratio may be decreased, which would likely reduce supernatant concentrations below regulatory limits. However, Pb remains of concern for direct ingestion, such as hand to mouth or swallowing inhaled particles, since the gastric PBET released a significant 37% of Pb to solution at concentrations in excess of 500 μg L^−1^. This labile pool of Pb may be due to high surface area and low particle size of Pb-bearing particles condensed from flue gases, consistent with the sub-micron, high average Z particles observed by SEM (Fig. S8). Prior work has reported Pb-oxides and sulfates in FA depending on combustion temperatures (Zhao et al. 2018), both forms with medium bioavailability (Ruby et al. 1999). Ubiquitous DOC in Alaskan surface waters makes PTE-DOM interactions an essential consideration. The lower supernatant Pb concentration when reacted with terrestrially derived DOM (SRFA), characterized by a higher fraction of aromatic moieties. AKSE DOM used in this study is also largely derived from terrestrial origin (although Pb not determined for this sample; Gagne et al. 2020). Thus, Pb released into an aquatic system dominated by terrestrially derived DOM should be less labile.

## Conclusions

Overall, leachate concentrations and the percent of PTEs liberated from the solid-phase FA were relatively low, with two notable exceptions: Pb and Ba. However, even after leaching for 90 days, many elements had apparently failed to reach equilibrium conditions. Low leaching rates relative to prior studies are likely explained by the high amount of residual organic material in the FA or the formation of neoformed minerals that incorporate or sorb PTEs, especially in the case of oxyanion-forming PTEs. Concomitant characterization of the supernatant and solid-phase transformations was essential to explaining leaching trends. Two examples of this being the high concentration of Ca in solution at 1 h and concurrent dissolution of calcite and the initial dissolution of ettringite followed by formation, which was inversely correlated with the highest concentrations of oxyanion-forming elements in solution. The role of unburned carbon in the release of PTEs is clearly important, especially in this FA with about 20% organic material, and may also play an important role in PTE release or sequestration. Additionally, the identity of DOM and initial DOC concentration affected the leaching behavior for some oxyanions. PTE-DOM interactions could be especially important for the fate of FA in the surficial Alaskan environment, since several elements had different extents of liberated species in the presence of AKSE relative to other DOM isolates. Although the specific mechanisms of how DOM identity and DOC concentrations influence PTEs is beyond the scope of a single study, it is clearly essential and requires additional scrutiny.

Storage and use of FA have the potential to introduce PTEs to the surficial environment and are a mechanism for PTE dispersion, which may adversely impact both environmental and human health. Despite low liberation rates, potentially toxic concentrations of PTEs were identified in both long-term and standard leaching procedures, but the standard procedures failed to characterize short- or long-term leaching trends. Further, several of the PTEs in FA were readily liberated under physiological conditions, highlighting the human health risk associated with these materials if inhaled or ingested. Additionally, the high solid:solution ratio used here maintained a high pH, thereby stabilizing minerals like ettringite that are stable at high pH and maintaining negatively charged organic moieties. However, in the surficial environment, these extreme conditions are not likely to persist over decadal timescales, and changing conditions may destabilize FA minerals to become a potential source of PTEs to the surficial environment.

## Supplementary information

ESM 1 (DOCX 5609 kb)

## Data Availability

All data generated or analyzed during this study either are included in this published article and its supplementary information files or are available in the ScienceBase repository, https://www.sciencebase.gov/catalogMaps/mapping/ows/5ffc9019d34e52c3b3d9d872?service=wms&request=getcapabilities&version=1.3.0.
