# Supplementary material for: Behavior of potentially toxic elements from stoker-boiler fly ash in Interior Alaska: paired batch leaching and solid-phase characterization
Source: Environ Sci Pollut Res Int. 2021 Oct 23;29(21):31059–74. doi: 10.1007/s11356-021-15583-x (PMC9054907; doi:10.1007/s11356-021-15583-x)
Supplement: Supplementary file 1 — (DOCX 5609 kb) [file 11356_2021_15583_MOESM1_ESM.docx]

**Supplementary Information:**

**Behavior of Potentially Toxic Elements from Stoker-Boiler Fly Ash in Interior Alaska: Paired Batch Leaching and Solid-Phase Characterization**

Kyle P. Milke,^1^ Kiana L. Mitchell,^1^ Sarah M. Hayes,^2^* Carlin J. Green,^2^ and Jennifer J. Guerard^3^

^1^ Department of Chemistry & Biochemistry, University of Alaska Fairbanks,

Fairbanks, AK 99775, United States

^2^ Geology, Energy & Minerals Science Center,

U. S. Geological Survey, Reston, VA 20192, United States

^3^ Chemistry Department, United States Naval Academy,

Annapolis, MD, 21402, United States

Submitted for publication in: *Environmental Science and Pollution Research*

***Contact information for corresponding author:** mailing: 12201 Sunrise Valley Dr., MS 954, Room 4C200 Reston, VA 20192; e-mail: shayes@usgs.gov, phone: (703) 648-6461, fax: (703) 648-6383

Total Pages: 24

**Figure S1:** Schematic of spreader stoker-boiler UAF power plant

**Figure S2**: Photographs of unreacted and ashed Usibelli coal and FA

**Figure S3:** X-ray diffraction patterns of unreacted and ashed Usibelli coal

**Figure S4:** X-ray diffraction patterns of unreacted and ashed FA

**Figure S5:** X-ray diffraction patterns of 14 d 18MΩ replicates

**Figure S6:** Scanning Electron microscopy of FA in a polished puck

**Figure S7:** Scanning Electron microscopy of FA dispersed on tape

**Table S1:** Detailed elemental analysis of fly ash and standard reference materials

**Table S2**: Munsell color of Usibelli coal and FA subjected to low and high temperature ashing

**Table S3:** Mineralogy of unreacted and ashed Usibelli coal

**Table S4:** Mineralogy of unreacted and ashed FA

**Table S5:** Mineralogy of unreacted FA and solid-phase residuals from 18MΩ and RW leaching experiments

**Table S6:** Mineralogy of 14 d 18MΩ replicates

**Table S7:** Synthetic precipitation leaching procedure (SPLP) supernatant concentrations

**Table S8:** Long-term batch leaching experiment supernatant concentrations

**Site description**

Samples examined in this study were sourced from the Atkinson Heat and Power Plant, a stoker-boiler system with a traveling grate at the University of Alaska Fairbanks (UAF). This facility has been operating since January 1964 and, at the time of collection, the plant generated 3,700 kW h^-1^ electricity and 100,000 lbs steam for building heat and hot water (**Fig. S1**). During combustion, coal pieces (passing a 5-cm grate) were fed from a storage hopper and distributed evenly across a traveling grate using a feed mechanism. Combustion occurs at temperatures in excess of 850 °C, with fine particles combusting while suspended in air and coarser particles being supported on the grate during combustion, as they travel toward a hopper where bottom ash is collected (Tomei 2005). Flue gasses produced during combustion travel into the cinder reinjection hopper, where heavier particles were reinjected into the furnace. Flue gases were further treated remove particulate matter using cyclone separators and a bag house prior to releasing gases back to the environment.

**Figure S1:** Schematic of spreader stoker-boiler UAF power plant.

**Elemental Composition**

*Solid phase*

Solid phase elemental analysis of FA was primarily performed by AGAT Laboratories (Mississauga, Canada). Briefly, major elemental composition was analyzed by wavelength-dispersive x-ray fluorescence (WD-XRF) after samples were fused into glass disks with lithium metaborate/lithium tetraborate flux. Total sulfur and total carbon by combustion followed by infrared detection using a LECO CHNS analyzer (Laboratory Equipment Corporation). Trace elemental analysis was performed using inductively coupled-optical emission spectrometry (ICP-OES) and inductively coupled-mass spectrometry (ICP-MS) following sample digestion in hot HCl, HNO_3_, HF, and HClO_4_ in Teflon vessels, and sintering of any residual solids with Na_2_O_2_ and NaOH. Matrix-matched NIST standard reference materials (NIST 2691 and NIST 1633a) were also measured in order to assess accuracy of the above elemental analyses. Quality assurance results were typically within 10-15% of certified or provisional values except at concentrations near the detection limit, as shown in **Table S1**. Solid-phase total carbon (TC) was analyzed in triplicate at the Cold Regions Research and Engineering Laboratory (CRREL, Fairbanks, AK) using a TOC-L analyzer (Shimadzu; Columbia, MD) equipped with a Solid Sample Combustion Unit (SSM-5000A). Five-point calibration was performed using anhydrous dextrose; potassium hydrogen phthalate quality control checks were performed every 10 analyses.

*Aqueous phase*

Elemental analysis of leachates was performed at the UAF Advanced Instrumentation Laboratory (AIL) on an Agilent 7500ce inductively-coupled plasma-mass spectrometer (ICP-MS; Santa Clara, CA) with appropriate internal (100 ppb Sc, Ge, Y, Rh, and Ir) and external calibration with 5-2000 µg kg^-1^ for major elements (Al, Ba, and sometimes Ca) and 0.1-100 µg kg^-1^ for minor elements (Fe, V, Cr, Mn, Co, Cu, Zn, As, Se, Mo, Sb, Pb, and Bi). Samples were quantitatively diluted to be within the calibration range. Trace metal-grade acid was used for all dilutions. Appropriate calibration and blank checks were performed every 10 samples, and standard reference waters were measured with every sample batch. Calcium was quantified using a flame atomic absorption (Perkin Elmer AA800; Waltham, MA) with appropriate external calibration (0.1-10 mg kg^-1^ Ca), sample dilution, blanks, and calibration checks. Detection limits were determined based on tuning and calibration, which were also used to validate calibration and standard checks throughout the run. All samples were blank subtracted (either acid blank or ½ detection limit, which ever was greater) and corrected for dilution. Acid blanks were measured and with the exception of Al and Ba, the concentrations of the acid blanks for the elements investigated never exceeded 1 ppb.

*Physical characterization*

Brunauer-Emmett-Teller surface area was measured by 3-point static volume gas absorption using a micrometrics TriStar II 3020 (Particle Technology Labs, Drowners Crove, IL). Stoker-boiler fly ash used in these experiments had a BET surface area of 89.64 m^2^ g^-1^. Low temperature ashing was performed to isolate the mineral fraction, followed by sequential heating to 250 °C, 400 °C, 550 °C, and 750 °C for at least 2 h in order to examine changes in mineralogy upon heating (e.g., the removal of structural waters and decomposition of carbonate minerals). Color was determined on dry samples using a Munsell soil color chart (**Fig. S2** and **Table S2**; Munsell Color; Grand Rapids, MI).

**Table S1:** Detailed elemental analysis of CCPs and quality control.

| **Elements** | **Fly Ash** | **SRM NIST 2691** | | |  | **SRM NIST 1633a** | | |
| --- | --- | --- | --- | --- | --- | --- | --- | --- |
|  |  | Meas. Value | Cert. Value^a^ | % Dif.^b^ |  | Meas. Value | Cert. Value^a^ | % Dif.^b^ |
| **Element (wt.%)** | | | | | | | | |
| **C** | 18.6 ± 0.1 | 0.09 |  |  |  | 2.77 |  |  |
| **S** | 2.12 ± 0.08 | 0.849 | 0.83 | 2 |  | 0.158 | 0.18 | -12 |
| **Al_2_O_3_** | 11.4 ± 0.2 | 18.3 | 18.54 | -1 |  | 27.2 | 27.02 | 1 |
| **BaO** | 0.55 ± 0.02 | 0.7 | 0.66 | 6 |  | 0.14 | 0.17 | -18 |
| **CaO** | 26.9 | 25.5 | 25.81 | -1 |  | 1.54 | 1.55 | -1 |
| **Cr_2_O_3_** | 0.02 | <0.01 | 0.01 |  |  | 0.03 | 0.03 | 0 |
| **Fe_2_O_3_** | 9.78 ± 0.01 | 6.29 | 6.32 | 0 |  | 13.5 | 13.44 | 0 |
| **K_2_O** | 0.64 ± 0.02 | 0.41 | 0.41 | 0 |  | 2.26 | 2.26 | 0 |
| **MgO** | 5.25 ± 0.01 | 5.22 | 5.17 | 1 |  | 0.75 | 0.75 | 0 |
| **MnO** | 0.36 ± .01 | 0.02 | 0.03 | -33 |  | 0.02 |  |  |
| **Na_2_O** | 0.17 ± 0.03 | 1.45 | 1.47 | -1 |  | 0.16 | 0.23 | -30 |
| **P_2_O_5_** | 0.12 ± 0.01 | 1.15 | 1.17 | -2 |  | 0.4 |  |  |
| **SiO_2_** | 18 ± 1 | 36.2 | 36.01 | 1 |  | 48.8 |  |  |
| **SrO** | 0.27 ± 0.01 | 0.37 | 0.32 | 16 |  | 0.09 | 0.1 | -10 |
| **TiO_2_** | 0.43 ± 0.01 | 1.53 | 1.5 | 2 |  | 1.39 | 1.33 | 5 |
| **V_2_O_5_** | 0.03 | 0.04 |  |  |  | 0.05 | 0.05 | 0 |
| **LOI** | 21.7 ± 0.1 | 0.31 | 0.23 | 35 |  | 3.45 |  |  |
| **Element (mg kg^-1^)** | | | | | | | | |
| **Al** | 6.28 | 9.81 | 9.81 | 0 |  | 14.8 | 14.3 | 3 |
| **Ca** | 19.00 | 17.6 | 18.45 | -5 |  | 1.19 | 1.11 | 7 |
| **Fe** | 6.38 ± 0.02 | 3.99 | 4.42 | -10 |  | 8.85 | 9.4 | -6 |
| **K** | 0.54 ± 0.01 | 0.36 | 0.34 | 6 |  | 1.95 | 1.88 | 4 |
| **Mg** | 3.18 ± 0.04 | 3.13 | 3.12 | 0 |  | 0.47 | 0.455 | 3 |
| **P** | 0.04 | 0.53 | 0.51 | 4 |  | 0.17 |  |  |
| **S** | 1.82 ± 0.02 | 0.77 | 0.83 | -7 |  | 0.16 | 0.18 | -11 |
| **Ti** | 0.24 | 0.85 | 0.9 | -6 |  | 0.82 | 0.8 | 2 |
| **Ag** | 2.10 | 1.4 |  |  |  | 1.3 |  |  |
| **As** | 124.5 ± 0.7 | 25 | 30 | -17 |  | 163 | 145 | 12 |
| **Ba** | 4320 ± 20 | 6120 | 5900 | 4 |  | 1330 | 1500 | -11 |
| **Be** | 3.05 ± 0.07 | 4.2 | 8 | -48 |  | 13.7 | 12 | 14 |
| **Bi** | 3.0 ± 0.2 | 0.8 |  |  |  | 1.1 |  |  |
| **Cd** | 5.8 ± 0.2 | 1.1 |  |  |  | 1.1 | 1 | 10 |
| **Ce** | 69 ± 1 | 103 |  |  |  | 165 | 180 | -8 |
| **Co** | 27.4 ± 0.6 | 26.4 | 26 | 2 |  | 47.8 | 46 | 4 |
| **Cr** | 85 | 58 | 68 | -15 |  | 185 | 196 | -6 |
| **Cs** | 4.9 ± 0.3 | 1.2 | 1 | 20 |  | 9.7 | 11 | -12 |
| **Cu** | 840 ± 20 | 192 |  |  |  | 116 | 118 | -2 |
| **Dy** | 6.23 ± 0.03 | 8.1 |  |  |  | 14 |  |  |
| **Er** | 3.6 ± 0.1 | 4.82 |  |  |  | 7.91 |  |  |
| **Elements** | **Fly Ash** | **SRM NIST 2691** | | |  | **SRM NIST 1633a** | | |
|  |  | Meas. Value | Cert. Value^a^ | % Dif.^b^ |  | Meas. Value | Cert. Value^a^ | % Dif.^b^ |
| **Element (mg kg^-1^)** | | | | | | | | |
| **Eu** | 2.31 ± 0.05 | 3.04 | 2 | 52 |  | 3.7 | 4 | -8 |
| **Ga** | 36 ± 2 | 30.5 |  |  |  | 59 | 58 | 2 |
| **Gd** | 7.62 ± 0.06 | 9.56 |  |  |  | 16.8 |  |  |
| **Ge** | 0.2 | 0.1 |  |  |  | 2.5 |  |  |
| **Hf** | 3.2 | 9 | 10 | -10 |  | 6.9 | 8 | -14 |
| **Hg** | 3.6 ± 0.1 | 0.08 | 0.058 | 38 |  | 0.188 | 0.16 | 18 |
| **Ho** | 1.30 ± 0.02 | 1.68 |  |  |  | 2.88 |  |  |
| **In** | 0.40 | <0.2 |  |  |  | <0.2 |  |  |
| **La** | 33.4 ± 0.4 | 56.2 |  |  |  | 78.2 |  |  |
| **Li** | 23.5 ± 0.7 | 47 |  |  |  | 207 |  |  |
| **Lu** | 0.49 ± 0.01 | 0.71 |  |  |  | 1.07 |  |  |
| **Mn** | 2670 ± 10 | 148 | 200 | -26 |  | 177 | 179 | -1 |
| **Mo** | 11.4 ± 0.3 | 8.9 |  |  |  | 31.9 | 29 | 10 |
| **Nb** | 7.4 ± 0.1 | 32.1 |  |  |  | 27.8 |  |  |
| **Nd** | 32.6 ± 0.4 | 46.2 |  |  |  | 75.4 |  |  |
| **Ni** | 125 | 52 | 53 | -2 |  | 121 | 127 | -5 |
| **Pb** | 140 ± 10 | 35.8 | 29 | 23 |  | 67.8 | 72.4 | -6 |
| **Pr** | 7.9 ± 0.1 | 11.8 |  |  |  | 18.9 |  |  |
| **Rb** | 34 ± 1 | 18.6 |  |  |  | 142 | 131 | 8 |
| **Sb** | 27 ± 2 | 2.5 |  |  |  | 6.9 |  |  |
| **Sc** | 17.6 ± 0.1 | 24.6 | 24 | 3 |  | 38.5 | 40 | -4 |
| **Se** | 22 | 13 | 17 | -24 |  | 11 | 10.3 | 7 |
| **Sm** | 7.1 | 9.1 |  |  |  | 15.8 |  |  |
| **Sn** | 8.0 ± 0.3 | 3.1 |  |  |  | 5.9 |  |  |
| **Sr** | 2160 | 2990 | 2700 | 11 |  | 803 | 830 | -3 |
| **Ta** | 0.5 | 1.9 |  |  |  | 1.9 |  |  |
| **Tb** | 1.08 ± 0.01 | 1.37 |  |  |  | 2.46 |  |  |
| **Te** | 0.65 ± 0.07 | 0.9 |  |  |  | 0.2 |  |  |
| **Th** | 11.8 ± 0.4 | 23.4 | 26 | -10 |  | 24.3 | 24.7 | -2 |
| **Tl** | 2.3 ± 0.1 | 0.6 |  |  |  | 5.3 | 5.7 | -7 |
| **Tm** | 0.51 ± 0.01 | 0.72 |  |  |  | 1.12 |  |  |
| **U** | 4.28 ± 0.06 | 7.75 |  |  |  | 9.27 | 10.2 | -9 |
| **V** | 160 ± 3 | 232 |  |  |  | 322 | 297 | 8 |
| **W** | 8.0 ± 0.4 | 2.7 |  |  |  | 5.8 |  |  |
| **Y** | 40.7 ± 0.1 | 53 |  |  |  | 91.4 |  |  |
| **Yb** | 3.35 ± 0.07 | 4.6 |  |  |  | 7.4 |  |  |
| **Zn** | 237 ± 8 | 96 | 120 | -20 |  | 232 | 220 | 5 |
| **Zr** | 114 ± 2 | 341 |  |  |  | 254 |  |  |

^a^ Certified values obtained from NIST certificates. Underlined values are provisional values.

^b^ Percent difference = [(measured value- certified value) divided by the certified value] *100 percent.

**Low and High Temperature Ashing Treatments and Mineralogy**

Healy coal and unreacted FA were subjected to low and high temperature ashing pretreatments after drying overnight at 100 ºC. Ashing treatments were performed to oxidize organic matter, volatilize or alter specific mineral phases to facilitate phase identification (Brindley and Brown 1980; Pike et al. 1989). Low temperature ashing (LTA) was performed using an oxygen plasma asher (LTA-504, LFE corporation, Waltham, MA) until constant mass was attained as determined by daily weighing. High temperature ashing was performed in pre-baked, pre-weighed, acid-washed ceramic crucibles by heating to 250 °C for 2 hours prior LTA treatment. Additional samples were heated sequentially to 250 °C, 400 °C, 550 °C, and 750 °C for at least 2 hours (See photos in **Fig. S2** and colors in **Table S2**).

XRD analysis was performed on: 1) each unreacted coal and FA, 2) each unreacted coal and FA subjected to low and high temperature ashing (**Figs. S3- S4** and **Tables S3-S4**), 3) solid-phase residuals from leaching experiments at selected timepoints (1 h, 2 d, 7 d, 14 d, 28 d, 90 d; **Fig. S5**, **Table S5**), and 4) experimental replicates of 14 d 18 MΩ experiment samples as well as a second analysis of one of the replicates (**Fig. S6**, **Table S6**). All FA samples were spiked with a corundum (Al_2_O_3_) internal standard at 15% wt/wt in order to quantify the fraction of amorphous material present (Brindley and Brown 1980). The sample-spike mixtures were micronized in isopropanol for 3 min, dried, and sieved with a 40-mesh brass sieve. All samples were then packed into 16-mm back-loaded sample holders. XRD analysis was performed using PANalytical X’Pert PRO (Almelo, The Netherlands) automated powder diffractometer with a 10-mm mask with Cu Kα radiation source operating at 45 kV and 40 mA. Samples were rotated at 8 rpm during the analysis while the angle was scanned from 3 to 80 °2θ at a step size of 0.0167° with a counting time of 200 s per point.

Quantitative mineral phase identification was performed using the Rietveld module of PANalytical X’Pert HighScore Plus software (Almelo, The Netherlands; version 4.8.0.25518) and standard reference patterns from the Inorganic Crystal Structure Database (ICSD; Fiz Karlsruhe, 2013). Based on prior work, Rietveld refinements are typically within + 3% of actual values, but may vary based on analytical conditions as well as the weight percent and nature of various phases present in a mixed matrix, especially the presence of amorphous material (Smith et al. 2013). However, these samples are known to contain a significant fraction of amorphous organic and glassy material, complicating absolute mineral abundance determinations. Despite this, changes in the relative percentage fits are considered to reflect mineralogical changes and additional sensitivity testing using experimental and analytical replicates. Depending on the peak position and peak overlaps, analysis of XRD patterns with Rietveld refinements typically has detection limits of 1% (Smith et al. 2013), but detection limits as low as ~0.2% have been reported in some systems (Leon-Reina et al. 2016). Some mineral phases are reported at values near and below this detection limit based on diagnostic peak positions and/or phase confirmation using SEM.

Rietveld fits were performed by searching for major phases for each pattern prior to converting those patterns to Rietveld phases for fitting. Background was fit using a Cheybshev background with initially the number of coefficients sufficient to get the low 2-theta peaks above the background, but eventually 5 coefficients for all patterns for consistency. Specimen displacement was then fit once and fixed prior to fitting the major phases, one at a time, using cell parameters and peak width, with split peak width for corundum, quartz, calcite, and ettringite. March-Dolasse parameter was also fit for clay minerals, ettringite and bassanite to account for any preferred orientation during packing that resulted in higher intensity of peaks at low degrees 2-theta. Minor phases were then added progressively and unit cell parameters were fit. The number of fit parameters was allowed to increase progressively (background, scale factors, cell parameters, and peak widths) until all parameters were varied as part of the final fit.

LTA treatment of coal resulted in an 82% mass loss and significant color change (reddish black to very pale brown), indicating effective oxidation of organic material (**Fig. S2** and **Table S2**). This organic material complicated XRD analysis due to high fraction of amorphous material that contributed to low signal and high background in the unreacted coal XRD pattern. Quartz, kaolinite and illite/muscovite were detected and the goodness of fit indicators were poor relative to the other patterns (**Fig. S3** and **Table S3**). After LTA, similar silicate minerals were observed, but a large fraction of the XRD pattern is attributed to bassanite (CaSO_4_ 0.5H_2_O), which has been reported to form during LTA from the oxidation of organic sulfur (Allen et al. 1986; Pike et al. 1989). Upon heating to 250 °C after LTA, which should drive off clay-bound waters, no significant changes to the diffraction pattern were observed. This was expected since hydrated clays were not identified in any of the samples. The small amount of mass lost can be attributed to bassanite dehydration to form anhydrite in the coal sample (a process that occurs between 200 and 275 °C; Deer et al. 1992). When samples were heated to 400 °C, intended to cause the collapse of expandable clay minerals (e.g., smectite and vermiculite). Expandable clays were not identified in any of the diffraction patterns, so the ~1% mass loss is attributed to basanite dehydration. As expected, at 550 °C the diffraction peaks for kaolinite in the coal sample disappeared, due to structural disordering (Brindley and Brown 1980). Calcite was also observed in this sample, which should volatilize upon heating to 750 °C (Brindley and Brown 1980). Overall, only 12% of the coal by mass persisted after heating to 750 °C.

FA mineralogy did not change during ashing treatments, although the percentage of minerals in the fits did increase as mass fraction of amorphous organic material decreased (**Fig. S4** and **Table S4**). LTA resulted in 18% mass loss, but only a minor change in color (very dark gray to dark grayish brown), possibly indicating incomplete removal of residual organic matter. During the heat treatments, FA color progressively lightened with the largest change occurring when heated to temperatures in excess of 400 °C (dark gray to light olive brown; **Fig. S2**). The 20% cumulative mass loss during heating to 750 °C was largely be attributed to loss of organics. Mineral volatilization may have also occurred, but is difficult to isolate due to the concomitant loss of organic matter throughout the heating process. As expected, the amorphous fraction decreases during the ashing treatments, as organic material not detectable by XRD was oxidized (estimated 70% in unreacted vs ~40% in the LTA and 750 °C residual). Residual amorphous material is expected due to the presence of poorly ordered residual organic material as well as the or formation of poorly crystalline glassy material during combustion.

**Figure S2:** Photographs of unreacted, and low and high temperature ashed Usibelli coal, and FA. These images show the color changes associated with the removal of organic material. Unreacted samples are black. Lighter colors, resulting from LTA or progressive heating indicate removal of organic matter.

**Table S2**: Munsell color of Usibelli coal and FA subjected to low and high temperature ashing.

|  | **Coal** | | **Fly ash** | |
| --- | --- | --- | --- | --- |
| **Unreacted** | 2.5YR 2.5/1 | reddish black | gley 1 3/N | very dark gray |
| **LTA** | 10YR 8/3 | very pale brown | 2.5Y 4/2 | dark grayish brown |
| **250 °C + LTA** | 5Y 4/1 | dark grey | 2.5Y 4/2 | dark grayish brown |
| **400 °C** | 5Y 2.5/2 | black | 5Y 4/1 | dark gray |
| **550 °C** | 2.5 Y 6/3 | light yellowish brown | 2.5Y 5/4 | light olive brown |
| **750 °C** | 10YR 7/4 | very pale brown | 2.5Y 5/3 | light olive brown |

**Figure S3:** X-ray diffraction patterns of unreacted, and low and high temperature ashed Usibelli coal. Minerals identified include: A- anhydrite, At- anatase, B- bassanite, Br- brownmillerite, Ca- calcite, I- illite/mica, K- kaolinite, and Q- quartz.

**Table S3:** Mineralogy of unreacted and ashed Usibelli coal. Changes in mass are also noted. These Rietveld fits are intended for comparison with other fits within this system and are likely not reflective of actual abundances present in the samples, thus values are intended to be informational.

|  | **Mineral** | **Composition** | **Unreacted** | **LTA** | **250 °C + LTA** | **400 °C** | **550 °C** | **750 °C** |
| --- | --- | --- | --- | --- | --- | --- | --- | --- |
|  | Rwp | | 10.1 | 6.7 | 7.1 | 6.3 | 5.7 | 6.6 |
|  | GOF | | 8.6 | 4.3 | 4.7 | 4.1 | 3.5 | 4.1 |
| **Silicates** | Quartz | SiO_2_ | 46 | 34 | 48 | 63 | 44 | 55 |
|  | Kaolinite | Al_2_Si_2_O_5_(OH)_4_ | 48 | 14 | 15 | 16 |  |  |
|  | Illite/muscovite-2M1 | KAl_2_(Al,Si_3_)O_10_(OH,F)_2_ | 7 | 6 | 7 | 17 | 11 | 10 |
| **Others** | Brownmillerite | Ca_2_(Fe,Al)_2_O_5_ |  |  |  |  |  | 4 |
|  | Anatase | TiO_2_ |  |  |  | 2 | 1 |  |
|  | Anhydrite | Ca(SO_4_) |  |  | 4 | 3 | 2 | 30 |
|  | Bassanite | Ca(SO_4_)·0.5H_2_O |  | 46 | 20 |  |  |  |
|  | Calcite | Ca(CO_3_) |  |  | 7 |  | 42 |  |
|  | Percent of original mass remaining at each step | | 100 | 18 | 17 | 16 | 13 | 12 |

**Figure S4:** X-ray diffraction patterns of unreacted, and low and high temperature ashed unreacted FA. Minerals identified include: A- akermanite-gehlenite, Br- brownmillerite, C*- corundum (added as an internal standard), Ca- calcite, E- ettringite, H- hematite, M- merwinite, Q- quartz.

**Table S4:** Mineralogy of unreacted and ashed FA. Corundum was added as an internal standard to estimate the percentage of amorphous material. These Rietveld fits are intended for comparison with other fits within this system and are likely not reflective of actual abundances present in the samples, thus values are intended to be informational.

|  | **Mineral** | **Composition** | **Unreacted** | **LTA** | **250 °C + LTA** | **400 °C** | **550 °C** | **750 °C** |
| --- | --- | --- | --- | --- | --- | --- | --- | --- |
|  | Rwp | | 4.7 | 4.6 | 4.2 | 4.6 | 4.1 | 4.3 |
|  | GOF | | 3.3 | 2.9 | 2.7 | 2.9 | 2.6 | 2.8 |
|  | Corundum spike added (wt%) | | 15.0 | 15.1 | 15.0 | 15.1 | 15.1 | 15.2 |
|  | % corundum fit | | 36 | 24 | 23 | 26 | 23 | 23 |
|  | Percent amorphous | | 68 | 42 | 42 | 50 | 40 | 39 |
| **Silicates** | Quartz | SiO_2_ | 4 | 5 | 5 | 7 | 7 | 7 |
|  | Gehlenite-akermanite | Ca_2_Al(Si,Al)_2_O_7_ / Ca_2_MgSi_2_O_7_ | 0.7 | 3 | 3 | 2 | 3 | 2 |
|  | Merwinite | Ca_3_Mg(SiO_4_)_2_ | 0.5 | 7 | 4 | 1 | 4 | 9 |
| **Other** | Brownmillerite | Ca_2_(Fe,Al)_2_O_5_ | 0.4 | 6 | 5 | 4 | 5 | 3 |
|  | Hematite | Fe_2_O_3_ | 1 | 2 | 2 | 2 | 2 | 8 |
|  | Periclase | MgO | 1 | 3 | 3 | 3 | 3 | 4 |
|  | Ettringite | Ca_6_Al_2_(SO_4_)_3_(OH)_12_·26H_2_O | 8 | 20 | 25 | 21 | 22 | 22 |
|  | Calcite | Ca(CO_3_) | 16 | 12 | 12 | 11 | 13 | 8 |
|  | Percent of original mass remaining at each step | | 100 | 82 | 83 | 92 | 82 | 80 |

**Figure S5**: X-ray diffraction patterns of unreacted and selected solid-phase leaching residues. For plotting, all patterns were normalized to corundum peak at 57.5 degrees 2-theta equal to 100. Minerals identified include: A- akermanite-gehlenite, B- bassanite, Br- brownmillerite, C*- corundum (added as an internal standard), Ca- calcite, E- ettringite, H- hematite, Gy-gypsum, M- merwinite, P- periclase, Q- quartz.

**Table S5**: Mineralogy of unreacted FA and solid-phase residuals from 18MΩ and RW leaching experiments. Diffraction patterns are shown in Figure S5 and the same data are plotted in Figure 2 of the main text. These Rietveld fits are intended for comparison with other fits within this system and are likely not reflective of actual abundances present in the samples, thus values are intended to be informational. Corundum was added as an internal standard to estimate the percentage of amorphous material.

|  |  |  | **Unreacted** | **18 MΩ** | | | | | | **RW** | | | | | |
| --- | --- | --- | --- | --- | --- | --- | --- | --- | --- | --- | --- | --- | --- | --- | --- |
|  | **Mineral** | **Composition** |  | **1 h** | **2 d** | **7 d** | **14 d** | **28 d** | **90 d** | **1 h** | **2 d** | **7 d** | **14 d** | **28 d** | **90 d** |
|  | Rwp | | 4.5 | 6.6 | 6.0 | 5.5 | 5.8 | 6.0 | 4.0 | 5.9 | 6.0 | 5.7 | 5.8 | 6.3 | 4.1 |
|  | GOF | | 3.2 | 2.9 | 2.7 | 2.5 | 2.6 | 2.7 | 2.6 | 2.7 | 2.7 | 2.6 | 2.6 | 2.8 | 2.7 |
| **Internal Standard** | Corundum spike added (wt%) | | 15.0 | 15.0 | 15.0 | 15.2 | 15.1 | 15.6 | 15.8 | 15.3 | 15.0 | 15.0 | 15.1 | 15.5 | 15.1 |
|  | % corundum fit | | 34 | 33 | 37 | 36 | 36 | 34 | 37 | 33 | 36 | 37 | 36 | 37 | 35 |
|  | Percent amorphous | | 66 | 64 | 70 | 68 | 69 | 64 | 69 | 63 | 69 | 69 | 69 | 69 | 68 |
| **Silicates** | Quartz | SiO_2_ | 4 | 7 | 6 | 5 | 6 | 7 | 5 | 7 | 6 | 6 | 5 | 6 | 5 |
|  | Gehlenite, akermanite | Ca_2_Al(Si,Al)_2_O_7_ / Ca_2_MgSi_2_O_7_ | 0.8 | 0.8 | 0.9 | 0.9 | 0.8 | 0.8 | 0.8 | 0.9 | 1.0 | 0.8 | 0.8 | 0.8 | 0.8 |
|  | Merwinite | Ca_3_Mg(SiO_4_)_2_ | 1 | 2 | 2 | 1 | 2 | 2 | 2 | 2 | 2 | 2 | 2 | 2 | 2 |
| **Oxides** | Brownmillerite | Ca_2_(Fe,Al)_2_O_5_ | 1 | 1 | 1 | 1 | 1 | 1 | 1 | 1 | 1 | 1 | 1 | 1 | 1 |
|  | Periclase | MgO | 1 | 2 | 1 | 1 | 1 | 1 | 1 | 2 | 1 | 1 | 1 | 0.8 | 0.8 |
|  | Magnetite | Fe_3_O_4_ | 0.5 | 0.4 | 0.3 | 0.5 | 0.5 | 0.4 | 0.4 | 0.6 | 0.5 | 0.4 | 0.4 | 0.3 | 0.5 |
|  | Hematite | Fe_2_O_3_ |  | 0.3 | 0.5 | 0.3 | 0.3 | 0.5 | 0.2 | 0.3 | 0.4 | 0.4 | 0.3 | 0.6 | 0.1 |
|  | Anatase | TiO_2_ |  | 0.2 | 0.4 |  | 0.3 | 0.4 | 0.1 | 0.3 | 0.3 | 0.3 | 0.3 | 0.5 | 0.1 |
| **Sulfates** | Ettringite | Ca_6_Al_2_(SO_4_)_3_(OH)_12_·26H_2_O | 9 | 9 | 4 | 5 | 5 | 10 | 10 | 7 | 5 | 2 | 4 | 4 | 9 |
|  | Bassanite | CaSO_4_·1/2H_2_O |  |  | 2 | 2 | 2 | 1 |  | 1 | 2 | 1 | 2 | 3 |  |
|  | Gypsum | CaSO_4_·2H_2_O |  |  |  | 0.3 | 0.5 | 0.3 | 0.7 |  |  | 0.4 | 0.6 | 0.5 | 0.5 |
| **Other** | Calcite | CaCO_3_ | 16 | 13 | 10 | 14 | 12 | 11 | 12 | 14 | 12 | 15 | 14 | 13 | 13 |
|  | Iron | Fe |  |  | 0.1 |  |  | 0.1 |  |  |  | 0.1 |  | 0.2 |  |

**Fly Ash Replicate Analysis**

In an effort to assess the robustness of mineralogical identification, sample heterogeneity, and variability between replicates, 14 d 18 MΩ experimental replicates were examined. Sample heterogeneity was assumed to be much larger than instrumental error based on the consistent positions of the peaks (+0.03 degrees 2-theta) in a reference Si disc measured with each batch of samples. Replicate 1 was analyzed twice by XRD with repacking between measurements, in order to estimate the variability of XRD results within a single replicate. Also, the mineralogy was determined for each of three replicates, in order to examine the variability between replicates at a single timepoint. Variability between replicates was used to qualitatively consider what changes in mineralogy are likely important in interpreting changes observed in the mineralogy for the time series leached FA patterns.

Based on visual inspection and Rietveld refinements of all four patterns (rep.1_1, rep. 1_2, rep. 2, and rep. 3; **Fig. S6** and **Table S6**) all patterns are quite similar to one another. The most similar patterns to one another are rep. 1_1 and rep. 1_2, with the most striking difference in the fit being amount of ettringite identified each pattern (4.7% and 2.1% in rep. 1_1 and rep. 1_2, respectively). This is consistent with the visible difference in the ettringite peak intensities at low degrees 2-theta (i.e., 9.0 and 15.8 degrees 2-theta). All 14 d patterns have similar amorphous content to one another (range: 68 to 70% estimated amorphous material). This amorphous content is slightly higher than the unreacted FA, but similar to the rest of the time series leached FA patterns (ranging from 68 to 70.3% estimated amorphous material, Table S5), with the notable exception of both 1 h and the 28 d 18 MΩ residue (63-64% estimated amorphous material). The mineralogy of all 14 d replicates is quite similar except that replicate 1 patterns have detectable bassanite and lower gypsum relative to replicates 2 and 3. Variable bassanite and gypsum content is also evident in the time series leached FA data (**Table S5**), indicating that metastable bassanite may precipitate and transform to gypsum such that either or both may be present in each leach solid phase residual. Further, bassanite is detected in many early time series XRD patterns, but completely transforms to the more thermodynamically stable gypsum by 90 d. The variability in abundance of all other mineral phases is small between the 14 d replicates.

**Figure S6:** X-ray diffraction patterns of 14 d 18 MΩ replicates. For plotting, all patterns were normalized to corundum peak at 57.5 degrees 2-theta equal to 100. Minerals identified include: A- akermanite-gehlenite, B- bassanite, Br- brownmillerite, C*- corundum (added as an internal standard), Ca- calcite, E- ettringite, Gy-gypsum, H- hematite, M- merwinite, P- periclase, Q- quartz.

**Table S6:** Mineralogy of 14 d, 18 MΩ replicates. Corundum was added as an internal standard to estimate the percentage of amorphous material.

|  | **Mineral** | **Composition** | rep. 1_1 | rep. 1_2 | rep. 2 | rep. 3 |
| --- | --- | --- | --- | --- | --- | --- |
|  | Rwp | | 5.8 | 6.5 | 6.7 | 6.7 |
|  | GOF | | 2.6 | 3.0 | 3.1 | 3.1 |
| **Internal Standard** | Corundum spike added (wt%) | | 15.1 | 15.1 | 15.1 | 15.2 |
|  | % Corundum fit | | 36 | 38 | 35 | 37 |
|  | Percent amorphous | | 69 | 70 | 68 | 70 |
| **Silicates** | Quartz | SiO_2_ | 6 | 6 | 7 | 6 |
|  | Gehlenite, akermanite | Ca_2_Al(Si,Al)_2_O_7_ / Ca_2_MgSi_2_O_7_ | 1 | 1 | 1 | 1 |
|  | Merwinite | Ca_3_Mg(SiO_4_)_2_ | 2 | 2 | 1 | 1 |
|  | Brownmillerite | Ca_2_(Fe,Al)_2_O_5_ | 1 | 1 | 1 | 1 |
| **Sulfates** | Ettringite | Ca_6_Al_2_(SO_4_)_3_(OH)_12_·26H_2_O | 5 | 2 | 3 | 3 |
|  | Bassanite | CaSO_4_·1/2H_2_O | 2 | 2 |  |  |
|  | Gypsum | CaSO_4_·2H_2_O | 0.5 | 0.5 | 2 | 2 |
| **Oxides** | Periclase | MgO | 1.0 | 0.8 | 0.8 | 0.9 |
|  | Magnetite | Fe_3_O_4_ | 0.5 | 0.5 | 0.4 | 0.4 |
|  | Hematite | Fe_2_O_3_ | 0.3 | 0.3 | 0.3 | 0.2 |
|  | Anatase | TiO_2_ | 0.3 | 0.3 | 0.2 | 0.2 |
|  | Calcite | CaCO_3_ | 12 | 13 | 15 | 16 |

**Scanning Electron Microscopy**

A polished puck was prepared using ~ 0.3g of unreacted FA mixed with a similar mass of TransOptic compression mounting compound (Buehler) and pressed at 360 ^o^F and 4000 psi for 15 minutes to prepare the sample-containing surficial layer prior to progressive mechanical polishing with 240, 400, 600, and 1200 grit papers. The polished puck was coated with 10 nm carbon using an EM ACE600 (Leica, Buffalo Grove, IL) prior to examination with a Hitachi SU5000 field emission scanning electron microscope (SEM) at the U. S. Geological Survey (Reston, VA). The instrument was equipped with an EDAX Octane Plus 30 mm^2^ silicon drift detector for energy dispersive spectroscopy (EDS). Observations were made using an accelerating voltage of 20 kV, a spot intensity ranging from 50 to 70, and a working distance of ~10 millimeters. The eZAF Smart Quant Results included in the EDS reports were automatically calculated using a “first principles” standardless quantitative method, meaning that the results were not compared to a sample of known composition and considerable uncertainties may exist depending on the sample conditions and instrument settings used. However, the EDS results were used primarily in a qualitative manner to identify unique phases (e.g., barite, organic material) and confirm that the observed spectra were compositionally consistent with phases identified by XRD (**Fig. S7**).

The FA is composed primarily of organic matter with a porous texture, spherical glassy particles that formed during combustion, angular mineral particles likely originating in the coal and entrained in the flue gasses, and aggregates of smaller particles that were not assigned to a specific phase. Organic particles have a distinctive texture and appearance (**Fig. S7a**) and may exceed 100 µm in size. The glassy phase occurs as spheres or angular particles that generally range from <5 to 100+ µm in size, and were often chemically zoned and/or porous (**Fig. S7b**). Much of the material was very fine-grained and appeared aggregated. Glass composition based on the 17 EDS analyses of homogeneous particles indicated that major elements include: O (39+4 %), Ca (21+7%), Si (18+4%), Al (11+4%), Fe(8+5%), Mg (2.5+0.8%), Ti (1+1%), and intermittently trace (<1 atomic %) amounts of Na, K, and Mn (all values listed in weight % and were semi-quantitative in nature; **Fig. S7c**). Many analyses contained O-Ca-Si-Fe-Al-Mg+K+Na+Ti, which were difficult to unambiguously identify as specific minerals, but the chemistry is consistent with the mineral suite identified by XRD. Based on EDS, minerals were tentatively identified, including residual organic matter, barite, elemental Fe (**Fig. S7c**) and possibly Fe (oxy)hydroxides, periclase, quartz (**Fig. S7a**), calcite, and brownmillerite (**Fig. S7b**). A few spot analyses were not consistent with XRD-determined minerals, likely due to low abundance, including an unidentified Ca-Fe-O phase and an O-Si-Al phase that consistently contained trace amounts of K and was tentatively identified as mullite since that has been reported in other similar systems (Affolter et al. 2011). These results are consistent with XRD analyses. Peaks corresponding to potentially toxic elements were not observed in any EDS spectra.

In a separate experiment, FA particles were dispersed on carbon tape and coated with roughly 2 nm Ir using a Denton Vacuum Desk V (Denton Vacuum, Moorestown, NJ) sputter coater prior to imaging with FEI Quanta 200 environmental scanning electron microscopy (ESEM; FEI Company, Hillsboro, OR) at University of Alaska Fairbanks Advanced Instrumentation Laboratory. Analysis was performed under high vacuum mode (pressure <1.0x10^-4^ torr) with variable excitation voltage (10-30 kV), spot size (2-5), and working distance (5-11 mm). Element mapping was performed using an energy dispersive IXRF Systems Model 550i X-ray detector (IXRF Systems, Austin, TX).

Results, shown in **Figure S8**, are similar to the polished pucks, with porous organic material (**Fig. 8a**) and glassy spheres (**Fig. 8b**). However, this sample preparation highlights the sub-micron bright particles, corresponding to high average-Z, and potentially containing PTEs.

**Figure S7:** Scanning electron microscope images and associated EDS spectra. Particle morphologies include: A) Residual organic particle with characteristic porous texture with a quartz particle in a pore; B) Porous, chemically zoned spheres; C) Angular glassy particle with iron inclusion; D) Central bright area is mixed Ba-containing particle. Elements are listed in decreasing order of abundance, based on semi-quantitative EDS spectra and only elements >5% abundance by weight are listed. Except in the case of organic material (B1), carbon is assumed to be contributed by the carbon coat or nearby organic material.

**
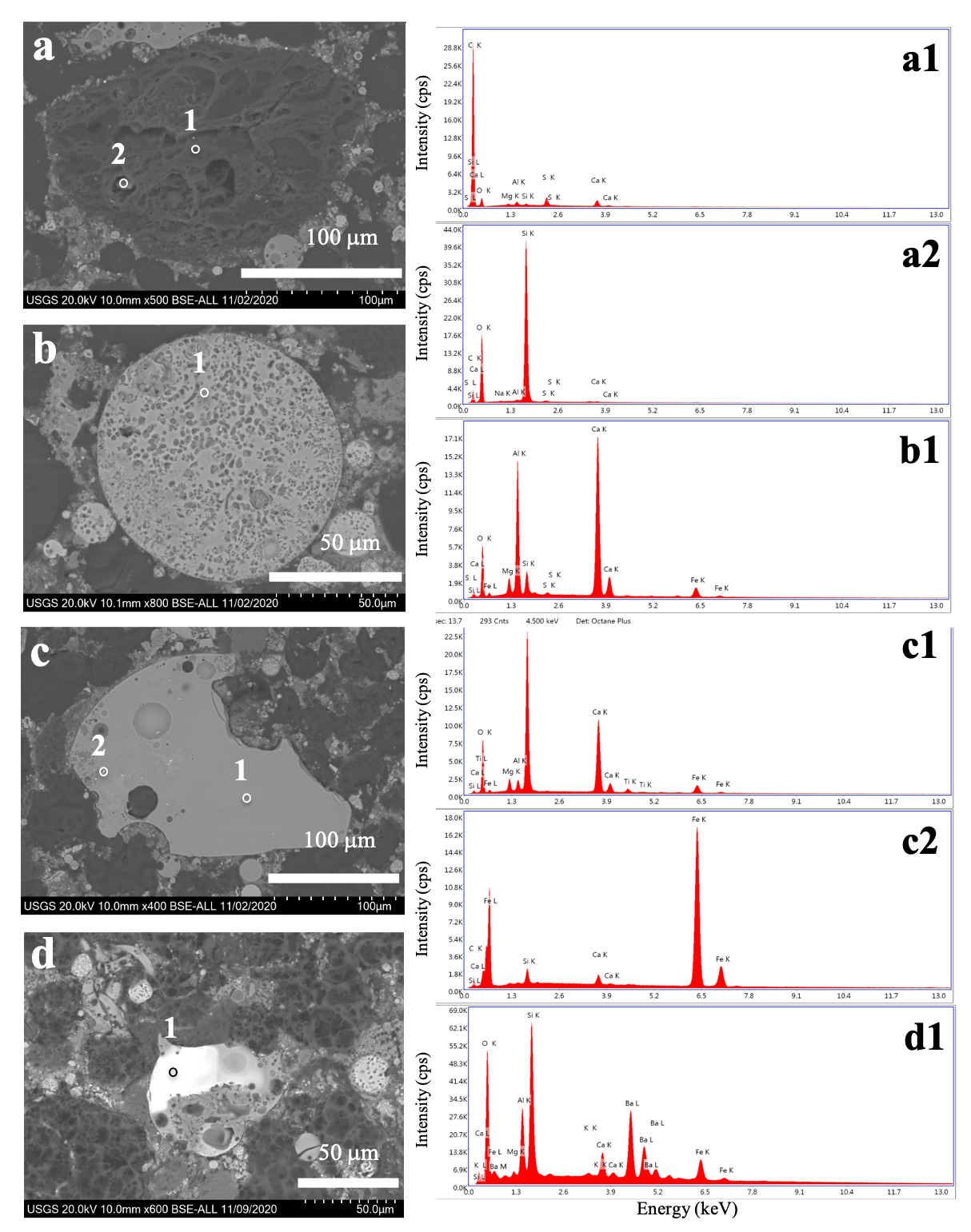
a1**: C-O-containing particle, consistent with organic matter.

**a2**: O-Si particle tentatively identified as quartz.

**b1**: O-Si-Ca-Fe-containing particle representing the glass phase.

**c1**: O-Ca-Al-Fe tentatively identified as brownmillerite.

**c2**: Fe-containing particle, tentatively identified as elemental iron.

**d1**: Ba-O-S-Al-Fe-containing particle, likely a mixture of barite and other mineral(s).

**Figure S8:** Scanning electron microscopy of FA dispersed on tape.

**
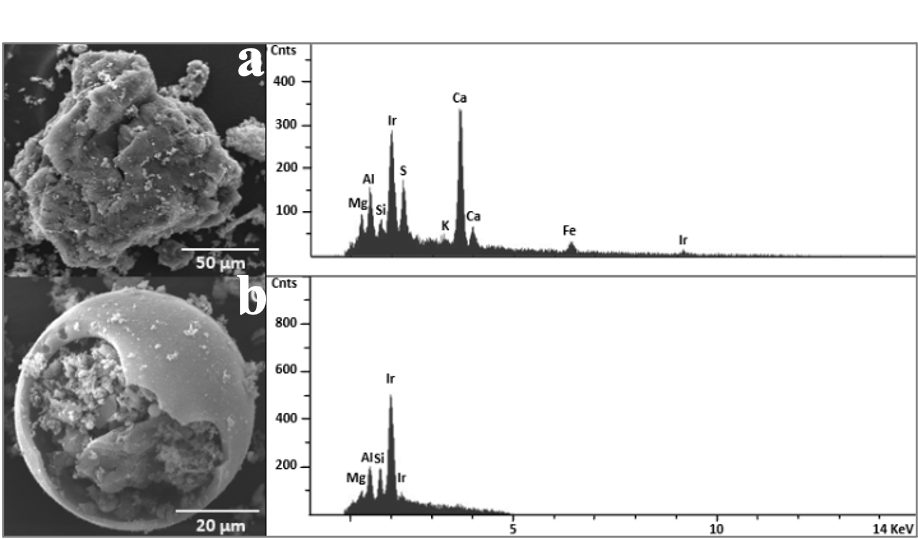
**

**Synthetic precipitation leaching procedure**

Synthetic precipitation leaching procedure (SPLP) was performed according to the EPA method 1312 (EPA 1994). Briefly, >18 MΩ cm^-1^ H_2_O was adjusted to pH 5.01 with a 60:40 mixture of concentrated H_2_SO_4_ and HNO_3_. Reagent and method blanks, and triplicate samples 50 g of FA were diluted to 1 L with the pH 5.01 solution and reacted on an end-over-end at 30 rpm for 18 h at room temperature. The samples settled for 2 h prior to filtration using acid-washed syringes, 0.2 µm polypropylene filters (Acrodisc GHP) and acidification to pH <2 for elemental analysis at UAF. Supernatant splits collected for analysis of pH, electrical conductivity (EC), alkalinity, and anions were filtered using new 0.45 µm hydrophilic polyethersulfone filters (Acrodisc Supor). The pH, EC, and alkalinity of the supernatants were measured immediately and anion samples were refrigerated prior to analysis. Alkalinity titrations were performed using phenolphthalein and total alkalinity determination by digital titration (Hach Company, Loveland, CO). Anion analysis was performed by ion chromatography using standard methods by the U.S. Geological Survey In-House Operational and Research Chemistry Laboratory (Denver, CO).

**Table S7:** Synthetic precipitation leach procedure supernatant parameters. Units are µg L^-1^, unless otherwise noted. EPA drinking water standards are also indicated (EPA 2018).

| **parameter** | **value** | **EPA Drinking Water MCL** | **parameter** | **value** | **EPA Drinking Water MCL** |
| --- | --- | --- | --- | --- | --- |
| pH | 12.39(1) |  | Zn | 8(1) |  |
| EC (ms cm^-1^) | 6.63(5) |  | As | 0.19(3) | 0, 10^c^ |
| Ca^a^ | 571(3)x10^3^ |  | Se | 1.03(4) | 50 |
| Ba^b^ | 26(3)x10^3^ | 2x10^3^ | Mo | 8(1) |  |
| Fe | 0.6(2) |  | Cd | 0.024(2) | 5 |
| Al^b^ | 900(100) |  | Sb | 0.74(6) | 6 |
| V | 0.31(6) |  | Pb | 29.5(9) | 0, 15^d^ |
| Cr | 0.12(3) | 100 | Cl^-^ | 0.79(3)x10^3^ |  |
| Mn | 4.1(4) |  | F^-^ | 5.46(6)x10^3^ | 4x10^3^ |
| Co | 1.11(2) |  | NO_3_^-^ | 0.93(6)x10^3^ | 1x10^4^ |
| Cu | 6.9(4) | 1.3x10^3 d^ | SO_4_^2-^ | 1.6(5)x10^3^ |  |
| Phenolphthalein Alkalinity (µg CaCO_3_ L^-1^) | | | | 1430(60)x10^3^ |  |
| Total Alkalinity (µg CaCO_3_ L^-1^) | | | | 1.51(4)x10^3^ |  |
| Hydroxide Alkalinity (µg CaCO_3_ L^-1^) | | | | 1.3(1)x10^3^ |  |
| Carbonate Alkalinity (µg CaCO_3_ L^-1^) | | | | 170(70)x10^3^ |  |

^a^ Calcium was measured by Flame AA, all other elements were measured by ICP-MS.

^b^ Measured value was above the highest calibration standard.

^c^ Maximum contaminant goal level (MCLG), maximum contaminant level (MCL) when not same value

^d^ Treatment Technique Action Level

**Physiological based extraction tests**

Physiological based extraction tests (PBETs), simulating the physiological conditions in the fasting stomach and lung conditions (EPA 2012; Stefaniak et al. 2006), were used to estimate the possible bioaccessibility of potentially toxic elements in unreacted FA. Simulated gastric fluid (SGF) was a 0.4 M L^-1^ glycine solution adjusted with OmniTrace HCl to pH = 1.5 (EPA 2012). The simulated lung fluid (SLF) was a freshly prepared modified Gambles solution at pH = 7.4 (Stefaniak et al. 2006). The PBET solutions were preheated to 37˚C prior to initiating the experiments. Triplicate experiments were performed with a ratio of 1 g FA to 100 mL SGF or SLF in an incubator shaker chamber (Lab-Line Instruments Inc., Melrose Park, IL) maintained at 37˚C for 1 h or 24 h for simulated gastric and lung fluids, respectively. The pH of the SGF samples was measured after 10, 30 and 45 minutes and adjusted, if necessary, with HCl. To terminate, samples were centrifuged, supernatant filtered and acidified prior to ICP-MS analysis for Cr, As, Se, Sb, and Pb.

PBET results indicate that, under physiological conditions, potentially toxic elements (e.g., Cr, As, Se, Sb, Pb) in the FA are solubilized (**Table 2**), many to a greater extent than pure water alone according to data from leaching experiments (**Fig. 3**, main text). EPA drinking water standards (**Table S7**) were exceeded by As, Sb, and Pb in simulated gastric conditions and by As and Se in simulated lung fluids. Higher concentrations were also solubilized in SGFs relative to SLFs, which is consistent with previous studies (Knight et al. 2017; Schaider et al. 2007). The exception to this is Se, which was solubilized to a higher degree by the SLF, likely due to the higher pH of the SLF extractant solution.

**Solubility plot**

Mineral dissolution reactions were used to model the saturation of several likely minerals in solution. Dissolution reactions (eq. 1-4) were used to develop mathematical relationships between barite and gypsum or ettringite at equilibrium, given that the sulfate ion concentrations would be the same if the phases are in equilibrium. Barite and gypsum solubility products were obtained from Skoog et al. 1996. Ettringite has a variety of published solubility products based on two different dissolution reactions (eq. 3-4), with two different aqueous Al species. The dissolution of ettringite with Al^3+^ product species (eq. 3) has published solubility product values of 10^-111.3^ and 10^-111.6 + 0.8^ in Atkins (1991; cited in Myneni et al. 1998) and Myneni et al*.* (1998), respectively. The dissolution of ettringite with Al(OH)_4_^-^ product species (eq. 4) has published solubility products of 10^-36^ (Jones 1944), 10^-45^ (Hampsoin and Bailey, 1982; cited in Myneni et al. 1998), 10^-44.55^ (Damidot and Glasser 1993; cited in Myneni et al. 1998), 10^-44.91^ (Warren and Reardon 1994), and 10^-44.90­+0.32^ (Perkins and Palmer 1999). Further, several oxyanions are known to substitute into ettringite, with the chromate and selenite endmembers being reported to have solubility products of 10^-41.46+0.3^ and 10^61.29+0.6^, respectively (Baur and Johnson 2003; Chrysochoou and Dermatas 2006; Perkins 2000). The zone of ettringite stability was calculated using the highest and lowest pH and concentration range of Al measured in sample solutions such that the largest stability zone was created. Further, the high and low solubility products (10^-45^ and 10^-36^) were similarly used to create the widest zone using eq. 4.

BaSO_4_ ⬄ Ba^2+^ + SO_4_^2-^ K_sp_ = 10^-9.96^ = [Ba^2+^] [SO_4_^2-^] (eq. 1)

CaSO_4_•2H_2_O ⬄ Ca^2+^ + SO_4_^2-^ +2 H_2_O K_sp_ = 10^-4.59^ = [Ca^2+^] [SO_4_^2-^] (eq. 2)

Ca_6_Al_2_(SO_4_)_3_(OH)_12_•26H_2_O ⬄ 6 Ca^2+^ + 2 Al^3+^ + 3 SO_4_^2-^ + 12 OH^-^ + 26 H_2_O

K_sp_ = [Ca^2+^]^6^ [Al^3+^]^2^ [SO_4_^2-^]^3^ [OH^-^]^12^ (eq. 3)

Ca_6_Al_2_(SO_4_)_3_(OH)_12_•26H_2_O ⬄ 6 Ca^2+^ + 2 Al(OH)_4_^-^ + 3 SO_4_^2-^ + 4 OH^-^ + 26 H_2_O

K_sp_ = [Ca^2+^]^6^ [Al(OH)_4_^-^]^2^ [SO_4_^2-^]^3^ [OH^-^]^4^ (eq. 4)

**Table S8:** Supernatant concentrations liberated from FA as a function of time (µg L^-1^).

| **Leachate: >18 MΩ cm^-1^ water (18 MΩ)** | | | | | | | | | | | | | | | | | | | | | |  |
| --- | --- | --- | --- | --- | --- | --- | --- | --- | --- | --- | --- | --- | --- | --- | --- | --- | --- | --- | --- | --- | --- | --- |
|  | **1 h** | **12 h** | | **24 h** | **48 h** | | **72 h** | | **5 d** | | **7 d** | | **10 d** | | **14 d** | | **21 d** | | **28 d** | | **90 d** | |
| pH | 12.77(1) | | 12.75(1) | 12.82(2) | 12.79(1) | 12.81(1) | | 12.89(3) | | 12.80(2) | | 12.85(2) | | 12.93 | | 12.91(1) | | 12.72(2) | | 12.41(1) | |  |
| Ca^a^ | 575(8)x10^3^ | | 600(20)x10^3^ | 620(20)x10^3^ | 644(9)x10^3^ | 680(10)x10^3^ | | 710(20)x10^3^ | | 700(20)x10^3^ | | 710(10)x10^3^ | | 690(20)x10^3^ | | 630(30)x10^3^ | | 580(30)x10^3^ | | 190(10)x10^3^ | |  |
| Ba | 30.0(5)x10^3^ | | 24(3)x10^3^ | 23(2)x10^3^ | 22(3)x10^3^ | 21(1)x10^3^ | | 20(3)x10^3^ | | 20(3)x10^3^ | | 22(3)x10^3^ | | 26(5)x10^3^ | | 29(3)x10^3^ | | 31(2)x10^3^ | | 8(2)x10^3^ | |  |
| Fe | 39(7) | | 70(10) | 60(20) | 71(4) | 70(10) | | 79(3) | | 70(5) | | 58(4) | | 143(3) | | 132(4) | | 126(4) | | 28.1(2) | |  |
| Al | 900(100) | | 620(70) | 450(30) | 400(100) | 273(5) | | 190(20) | | 250(40) | | 350(30) | | 400(80) | | 620(50) | | 1.4(2)x10^3^ | | 10.30(2)x10^3b^ | |  |
| V^c^ | 0.35(6) | | BDL | 0.25(5) | BDL | 0.47(2) | | 0.2(2) | | 0.45(7) | | 0.44(6) | | BDL | | 0.15(3) | | 0.22(4)1 | | 2.0(4) | |  |
| Cr^c^ | 0.11(3) | | BDL | BDL | BDL | BDL | | BDL | | BDL | | BDL | | BDL | | BDL | | BDL | | BDL | |  |
| Mn | 1.07(8) | | 1.1(7) | 1.7(2) | 0.9(1) | 2.1(2) | | 1.4(8) | | 1.4(4) | | 1.5(3) | | 0.7(2) | | 0.87(4) | | 1.1(1) | | 0.464(4) | |  |
| Co | 1.20(1) | | 1.24(3) | 1.24(5) | 1.31(1) | 1.41(3) | | 1.5(2) | | 1.54(4) | | 1.58(4) | | 1.83(5) | | 1.68(6) | | 1.58(7) | | 0.598(6) | |  |
| Cu | 12(2) | | 9(5) | 11(3) | 4(2) | 15(1) | | 12(8) | | 15(2) | | 16(1) | | 4(5) | | 6(1) | | 6(1) | | 0.5(1) | |  |
| Zn | 8.4(7) | | 5(2) | 4(1) | 3(1) | 5.1(5) | | 4(3) | | 5(1) | | 6(1) | | 10(2) | | 10(1) | | 11(1) | | 5(1) | |  |
| As^c^ | BDL | | BDL | BDL | BDL | 0.37(8) | | BDL | | 0.39(7) | | 0.29(3) | | BDL | | BDL | | BDL | | 0.366(3) | |  |
| Se | 0.8(3) | | 1.2(3) | 0.9(4) | 1.2(3) | 1.5(2) | | 1.4(3) | | 1.40(8) | | 1.3(1) | | 1.0(3) | | 0.9(2) | | 1.0(2) | | 1.9(1) | |  |
| Mo | 11(2) | | 13.6(8) | 11(1) | 13(3) | 15.2(7) | | 12.5(5) | | 13(1) | | 11.6(3) | | 8.4(6) | | 6.5(3) | | 7.2(7) | | 13(2) | |  |
| Sb | 1.0(2) | | 1.06(4) | 0.80(5) | 0.9(1) | 1.13(4) | | 0.70(8) | | 0.89(4) | | 0.87(3) | | 0.8(2) | | 0.92(1) | | 1.10(2) | | 0.6(1) | |  |
| Pb | 68.0(4)^d^ | | 42(1)^d^ | 36(1)^d^ | 36(2)^d^ | 41.6(5)^d^ | | 34(5)^d^ | | 38(5)^d^ | | 40(2)^d^ | | 22(2) | | 19(1) | | 19(2) | | 2.9(1) | |  |
| Bi | 0.045(3) | | 0.025(4) | 0.023(1) | 0.015(2) | 0.024(2) | | 0.0194(7) | | 0.015(2) | | 0.024(2) | | 0.492(3) | | 0.491(1) | | 0.492(2) | | 0.1561(3) | |  |

**Table S8:** Continued.

| **Leachate: Simulated rain water (RW)** | | | | | | | | | | | | | | | | | | | | | |  |  |
| --- | --- | --- | --- | --- | --- | --- | --- | --- | --- | --- | --- | --- | --- | --- | --- | --- | --- | --- | --- | --- | --- | --- | --- |
|  | | **1 h** | | **12 h** | | **24 h** | | **48 h** | **72 h** | **5 d** | | **7 d** | | **10 d** | | **14 d** | | **21 d** | | **28 d** | | **90 d** | |
| pH | | 12.69(3) | | 12.64(1) | | 12.77(2) | 12.73(1) | 12.78(2) | 12.80(2) | 12.78(1) | | 12.78(3) | | 12.83(5) | | 12.86(1) | | 12.71(1) | | 12.47(2) | |  |  |
| Ca^a^ | | 570(10)x10^3^ | | 560(6)x10^3^ | | 587(7)x10^3^ | 630(5)x10^3^ | 650(30)x10^3^ | 640(30)x10^3^ | 650(10)x10^3^ | | 620(20)x10^3^ | | 570(50)x10^3^ | | 540(8)x10^3^ | | 520(30)x10^3^ | | 200(20)x10^3^ | |  |  |
| Ba | | 37(2)x10^3^ | | 28(2)x10^3^ | | 26(2)x10^3^ | 22.6(6)x10^3^ | 24(1)x10^3^ | 20.8(7)x10^3^ | 23(2)x10^3^ | | 23(2)x10^3^ | | 21(2)x10^3^ | | 27(1)x10^3^ | | 27(2)x10^3^ | | 8.4(4)x10^3^ | |  |  |
| Fe | | 51.0(4) | | 53(2) | | 54(4) | 59.8(9) | 58(3) | 54(7) | 59(6) | | 55(2) | | 74(9) | | 77(7)1 | | 78(8) | | 12.9(6) | |  |  |
| Al^c^ | | 900(100) | | 590(50) | | 450(50) | 270(20) | 250(40) | 180(30) | 280(50) | | 430(70) | | 600(100) | | 1.1(1)x10^3^ | | 2.0(2)x10^3^ | | BDL | |  |  |
| V^c^ | | 0.3(3) | | BDL | | 0.2(2) | 0.60(2) | 0.4(3) | 0.61(6) | 0.4(3) | | 0.57(5) | | BDL | | 0.05(1) | | 0.11(7) | | 1.5(3) | |  |  |
| Cr^c^ | | 0.18(2) | | 0.21(4) | | 0.17(7) | 0.25(9) | 0.1(1) | 0.3(3) | 0.09(4) | | BDL | | BDL | | BDL | | BDL | | BDL | |  |  |
| Mn^c^ | | 0.4(3) | | BDL | | 0.9(3) | 0.9(2) | 0.61(9) | 0.4(2) | 0.5(2) | | 0.8(1) | | BDL | | 0.39(6) | | 0.7(2) | | 0.60(4) | |  |  |
| Co | | 1.04(6) | | 1.0(2) | | 1.089(8) | 1.20(1) | 1.22(5) | 1.25(8) | 1.24(2) | | 1.25(5) | | 0.7(1) | | 0.77(5) | | 0.70(3) | | 0.69(5) | |  |  |
| Cu^c^ | | 7(5) | | 4(6) | | 5(2) | 9.9(2) | 8(2) | 8(2) | 6(3) | | 8(1) | | BDL | | 2.5(5) | | 3(1) | | 0.6(2) | |  |  |
| Zn | | 14.9(3) | | 9(2) | | 9(1) | 9.5(3) | 9.1(2) | 8.4(2) | 8(1) | | 8.4(7) | | 5(1) | | 7.5(4) | | 8.0(4) | | 3(1) | |  |  |
| As | | 0.3(2) | | 0.2(3) | | 0.2(1) | 0.60(3) | 0.4(2) | 0.6(2) | 0.3(2) | | 0.42(5) | | BDL | | BDL | | BDL | | 0.18(3) | |  |  |
| Se | | 1.6(5) | | 1.9(5) | | 1.46(8) | 2.0(2) | 2.1(5) | 1.8(3) | 1.62(6) | | 1.55(4) | | 1.4(3) | | 1.7(5) | | 2.2(3) | | 1.8(3) | |  |  |
| Mo | | 11(2) | | 14(2) | | 11(1) | 16.0(7) | 15(2) | 14(2) | 12(1) | | 11(2) | | 9.26(8) | | 7.7(4) | | 8.2(4) | | 13(2) | |  |  |
| Sb | | 0.8(4) | | 0.8(2) | | 0.72(4) | 0.98(4) | 0.90(4) | 0.75(6) | 0.70(7) | | 0.8(1) | | 0.1027(1) | | 0.1023(9) | | 0.1026(2) | | 0.77(9) | |  |  |
| Pb | | 57(2) | | 28(5) | | 26(1) | 28.5(4) | 28.4(3) | 23(3) | 25(1) | | 24(3) | | 19(4) | | 19.1(5) | | 21(3) | | 3.9(7) | |  |  |
| Bi | | 0.05(1) | | 0.02(1) | | 0.018(2) | 0.031(5) | 0.021(2) | 0.016(1) | 0.021(5) | | 0.021(2) | | 0.445(3) | | 0.449(3) | | 0.453(4) | | 0.190(2) | |  |  |

^a^ Calcium was measured by Flame AA, all other elements were measured by ICP-MS.

^b^ Measured value was above the highest calibration standard.

^c^ Measured values below detection limits.

^d^ Measured calibration check and blanks were within acceptance criteria, but standard reference water NIST 1640A values were low (20-24 RSD).

**References**

Affolter RH, Groves S, Betterton WJ et al., (2011) Geochemical database of feed coal and coal combustion products (CCPs) from five power plants in the United States Data Series 635. https://pubs.usgs.gov/ds/635/

Allen R, Carling R, VanerSande J (1986) Microstructural changes in coal during low temperature ashing. Fuel 65:321-326. https://doi.org/10.1016/0016-2361(86)90290-5

Atkins M (1991) Solubility properties of ternary and quaternary compounds in the CaO- Al_2_O_3_-SO_3_-H_2_O system. Cem Concr Res 21:991-998. https://doi.org/10.1016/0008-8846(91)90058-P

Baur I, Johnson CA (2003) The solubility of selenate-AFt (3CaO·Al_2_O_3_·3CaSeO_4_·37.5H_2_O) and selenate-AFm (3CaO·Al_2_O_3_·CaSeO_4_·xH_2_O). Cem Concr Res 33:1741–1748. https://doi.org/10.1016/S0008-8846(03)00151-0

Brindley GW, Brown G (1980) Crystal structures of clay minerals and their X-ray identification. Mineralogical Society, London

Chrysochoou M, Dermatas D (2006) Evaluation of ettringite and hydrocalumite formation for heavy metal immobilization: Literature review and experimental study. J Haz Mat 136:20-33. https://doi.org/10.1016/j.jhazmat.2005.11.008

Damidot D, Glasser FP (1993) Thermodynamic investigation of the CaO- Al_2_O_3_-CaSO_4_-H_2_O system at 25°C and the influence of Na2O. Cem Concr Res 23:221-238. https://doi.org/10.1016/0008-8846(93)90153-Z

Deer W, Howie R, Zussman J (1992) An introduction to the rock forming minerals. 2nd edn. Longman Group Limited, Hong Kong

EPA, U. S. Environmental Protection Agency (1994) Method 1312: Synthetic precipitation leaching procedure EPA Method 1312. https://www.epa.gov/sites/production/files/2015-12/documents/1312.pdf

EPA, U. S. Environmental Protection Agency (2012) Standard operating procedure for an in vitro bioaccessibility assay for lead in soil EPA 9200.2-86. https://nepis.epa.gov/Exe/tiff2png.cgi/P100GESL.PNG?-r+75+-g+7+D%3A%5CZYFILES%5CINDEX%20DATA%5C11THRU15%5CTIFF%5C00000451%5CP100GESL.TIF

EPA, U. S. Environmental Protection Agency (2018) National primary drinking water regulations. https://www.epa.gov/ground-water-and-drinking-water/national-primary-drinking-water-regulations#one. Accessed 8-13-2019

Hampsoim CJ, Bailey JE (1982) On the structure of some precipitated calcium alumino-sulphate hydrates. J Mat Sci 17:3341-3346. https://doi.org/10.1007/BF01203504

Fiz Karlsruhe (2013) Inorganic Crystal Structure Database (ICSD), version 3.3. https://www.fiz-karlsruhe.de/en/produkte-und-dienstleistungen/inorganic-crystal-structure-database-icsd

Jones FE (1944) The quaternary system CaO– Al_2_O_3_–CaSO_4_–H_2_O at 25°C. Equilibria with crystalline Al_2_O_3_–3H_2_O, alumina gel, and solid solution. J Phys Chem 48:311-356. https://doi.org/10.1021/j150438a001

Knight DC, Ramos NA, Iceman CR, Hayes SM (2017) Is unpaved road dust near Fairbanks, Alaska a health concern? Examination of the total and bioaccessible metalloids. J Young Invest 33. https://doi.org/10.22186/jyi.33.1.7-17

Leon-Reina L, Garcia-Mate M, Alvarez-Pinazo G, Santacruz I, Vallcorba O, De la Torre AG, Aranda MA (2016) Accuracy in Rietveld quantitative phase analysis: a comparative study of strictly monochromatic Mo and Cu radiations. J Appl Crystallogr 49:722-735. https://doi.org/10.1107/S1600576716003873

Myneni S, Traina S, Logan T (1998) Ettringite solubility and geochemistry of the Ca(OH)_2_–Al_2_(SO_4_)_3_–H_2_O system at 1 atm pressure and 298 K. Chem Geol 148:1-19. https://doi.org/10.1016/S0009-2541(97)00128-9

Perkins RB (2000) The solublity and thermodynamic properties of ettringite, it’s chromium analogs, and calcium aluminum monochromate (3CaO- Al_2_O_3_-CaCrO_4_ nH_2_O). Portland State University

Perkins RB, Palmer CD (1999) Solubility of ettringite (Ca_6_[Al(OH)_6_]_2_(SO_4_)_3_ · 26H_2_O) at 5–75°C. Geochim Cosmochim Acta 63:1969-1980. https://doi.org/10.1016/S0016-7037(99)00078-2

Pike S, Dewison M, Spears D (1989) Sources of error in low temperature plasma ashing procedures for quantiative mineral analysis of coal ash. Fuel 68:664-668. https://doi.org/10.1016/0016-2361(89)90170-1

Schaider LA, Senn DB, Brabander DJ, McCarthy KD, Shine JP (2007) Characterization of zinc, lead, and cadmium in mine waste: Implications for transport, exposure, and bioavailability. Environ Sci Technol 41:4164-4171. https://doi.org/10.1021/es0626943

Skoog DA, West DM, Holler FJ (1996) Fundamentals of analytical chemistry. Saunders College Publishing, New York

Smith DB, Cannon WF, Woodruff LG, Solano F, Kilburn JE, Fey DL, US Geological Survey (2013) Geochemical and mineralogical data for soils of the conterminous United States U. S. Geological Survey Data Series 801. https://pubs.usgs.gov/ds/801/

Stefaniak AB, Day GA, Hoover MD, Breysse PN, Scripsick RC (2006) Differences in dissolution behavior in a phagolysosomal simulant fluid for single-constituent and multi-constituent materials associated with beryllium sensitization and chronic beryllium disease. Toxicol In Vitro 20:82-95. https://doi.org/10.1016/j.tiv.2005.06.031

Warren CJ, Reardon EJ (1994) The solubility of ettringite at 25°C. Cem Concr Res 24:1515-1524. https://doi.org/10.1016/0008-8846(94)90166-X
